# Stage-specific metabolic divergence in flavonoid biosynthesis correlates with embryogenic capacity in rubber tree (*Hevea brasiliensis*)

**DOI:** 10.3389/fpls.2026.1766162

**Published:** 2026-02-04

**Authors:** Jia Miao, Xiao-Long Sun, Jin Liu, Ming-Chun Gui, Min Tang, Hai Tian, Wan-Yuan Shi, Ling Li

**Affiliations:** Yunnan Key Laboratory of Sustainable Utilization Research on Rubber Tree, The Center of Rubber Research, Yunnan Institute of Tropical Crops, Jinghong, Xishuangbanna, China

**Keywords:** flavonoid biosynthesis, *Hevea brasiliensis*, metabolomics, somatic embryogenesis, transcriptomics

## Abstract

**Introduction:**

Somatic embryogenesis (SE) is an essential propagation technology for *Hevea brasiliensis*, yet its application remains limited by the strong genotype dependence of embryogenic capacity.

**Methods:**

To elucidate the metabolic basis of this variation, we conducted integrated metabolomic and transcriptomic analyses across four SE developmental stages in a high-embryogenic (HE) and a low-embryogenic (LE) genotype, including explants, induced callus, non-embryogenic / embryogenic callus, and cotyledonary embryos (HE-specific).

**Results:**

A total of 1,383 metabolites belonging to 11 major classes were identified, with flavonoids, phenolic acids, and amino acids being the predominant groups. PCA and hierarchical clustering revealed that metabolic variation was driven primarily by developmental stage rather than genotype. Differential metabolite profiling revealed strong stage specificity, with the callus-to-differentiation transition (LE-C vs. HE-EC) exhibiting the greatest metabolic divergence between genotypes. KEGG enrichment consistently highlighted flavonoid biosynthesis as a key differentiating pathway. Comparative analyses revealed a conserved-to-divergent pattern of metabolic regulation. During the explant-to-callus transition, both genotypes exhibited highly conserved flavonoid biosynthesis responses, with 67.5% of genes and 85.7% of metabolites showing concordant regulation (either both up-regulated or both down-regulated). In contrast, during the callus-to-differentiation transition, pronounced metabolic divergence emerged, with only 37.5% of genes and 6.7% of metabolites showing concordant regulation, and 11 flavonoid-related genes displaying opposite regulatory directions between genotypes. Notably, the HE genotype exhibited coordinated repression of *CHS, CHI, F3H, UFGT*, and anthocyanin biosynthesis, accompanied by decreased accumulation of naringenin and glycosylated flavonoids, along with an overall attenuation of dihydroflavonol accumulation. Conversely, the LE genotype maintained relatively active flavonoid biosynthesis and glycosylation, along with increased amino sugar and nucleotide sugar metabolism.

**Discussion:**

Our results provide comprehensive metabolomic evidence for stage-dependent metabolic reprogramming during SE in *H. brasiliensis*. The contrasting patterns of flavonoid metabolism between genotypes at the callus-to-differentiation transition—systematic downregulation in the HE genotype versus sustained activation in the LE genotype—are consistent with the hypothesis that a timely reallocation of metabolic flux from secondary to primary metabolism may favor somatic embryo development. This study identifies the callus-to-differentiation transition as a critical metabolic checkpoint and suggests flavonoid biosynthesis genes, particularly *CHS* and glycosyltransferases, as potential targets for improving SE efficiency in recalcitrant genotypes.

## 1 Introduction

Natural rubber is an essential industrial raw material whose unique polymer structure confers exceptional elasticity, resilience, and durability, making it irreplaceable in aerospace, medical, and automotive manufacturing (Van Beilen and Poirier, 2007; Cornish, 2017). *H. brasiliensis* is the only plant species capable of producing natural rubber at a commercially viable scale. Although native to the Amazon Basin, it is now predominantly cultivated in Southeast Asia (Metcalfe, 1967; Zhou et al., 2017; Cherian et al., 2019).

Effective propagation of *H. brasiliensis* remains challenging due to its highly heterozygous genome, long juvenile period, strong inbreeding depression, cross-pollinating reproductive system, recalcitrant seeds, and low germination rates (Clément-Demange et al., 2007). Conventional propagation largely depends on grafting. However, scion-rootstock interactions can substantially affect growth vigor and latex yield (Ahmad, 1999; Sobhana et al., 2001). Using heterogeneous seedlings as rootstocks often results in significant performance variation within the same clone (Li et al., 2016).

SE has emerged as a pivotal biotechnological tool for rapid clonal propagation and genetic transformation of *H. brasiliensis* (Hua et al., 2010; Li et al., 2015). Plants regenerated via SE retain juvenile traits and develop autonomous root systems, forming self-rooted juvenile clones (SRJCs). Compared to conventional grafted plants, SRJCs exhibit marked advantages in growth rate, rubber yield, stress tolerance, and uniformity (approximately 20% improvement in both growth and yield), positioning them as ideal planting material for future rubber cultivation (Wang et al., 1980; Carron and Enjalric, 1982; Hua et al., 2010; Mignon and Werbrouck, 2018).

SE can occur via two distinct pathways: a direct route, in which somatic embryos differentiate directly from explant tissues, and an indirect route, in which embryos arise through an intermediate callus phase. Through the indirect pathway, multiple explant-based SE systems have been successfully established in *H. brasiliensis*, including those derived from immature anthers, inner seed integuments, inflorescences, and axillary buds (Carron and Enjalric, 1982; Carron et al., 1992; Kala et al., 2009; Hua et al., 2010). Based on the somatic embryos generated from these systems, cyclic secondary somatic embryogenesis has been developed, enabling the continuous and theoretically unlimited production of somatic embryos. This cyclic SE technology provides the foundation for the large-scale clonal propagation of *H. brasiliensis* (Hua et al., 2010; Mignon and Werbrouck, 2018).

Despite these advances, SE in *H. brasiliensis* remains severely constrained by strong genotype dependence. Only a few genotypes (e.g., Yunyan73477, CATAS73397, CATAS917, and PB260) exhibit high embryogenic capacity and are suitable for large-scale propagation or transformation (Huang et al., 2019; Wang et al., 2023). In contrast, many commercially important cultivars, including Reken628 and CATAS879, display low or non-embryogenic capacity under existing culture conditions (Li et al., 2023; Wang et al., 2023). This genotype dependence causes dramatic variation in callus induction rate, embryo formation, and regeneration efficiency, even among callus lines derived from the same clone. Understanding the mechanisms underlying genotype-dependent SE competence is therefore critical for overcoming this bottleneck.

Over the past decade, substantial progress has been made in elucidating the molecular mechanisms regulating SE in plants. Transcriptomic analyses have identified key transcription factors—including Baby Boom (BBM), Leafy Cotyledon (LEC), WUSCHEL (WUS), and Growth-Regulating Factor (GRF)—as well as SE-related receptor-like kinases (SERKs) and late embryogenesis abundant (LEA) proteins that play essential roles in somatic embryo induction and development (Florez et al., 2015; Horstman et al., 2017; Shivani et al., 2017; Zhang et al., 2025). These studies collectively reveal that SE is governed by intricate interactions among transcriptional regulation, phytohormone signaling, and epigenetic modifications.

In *H. brasiliensis*, transcriptomic studies have identified DEGs associated with early embryogenic events, including MADS-box genes and AP2/ERF transcription factors (Wang et al., 2021). Recent functional studies further demonstrated that overexpression of HbGRF4 or HbGRF4-HbGIF1 chimeras significantly enhances SE efficiency in rubber tree callus (Luo et al., 2024). However, most studies have focused on individual genotypes or transcriptional regulation alone, leaving the metabolic basis of genotype-dependent embryogenic competence largely unexplored.

Metabolomics provides a direct readout of biochemical activity and represents the closest molecular layer to phenotype. In SE systems, metabolomic profiling has revealed key pathways associated with embryogenic potential in several species, including Norway spruce (Businge et al., 2012), coffee (Awada et al., 2019), and tea (Awon et al., 2024). These studies highlight critical roles of phytohormones (e.g., auxins, cytokinins, abscisic acid) and secondary metabolites (e.g., phenolics, flavonoids, amino acids) in controlling SE. Notably, excessive accumulation of flavonoids and phenolic compounds has been reported as a feature of non-embryogenic callus in several conifers, suggesting that aberrant activation of secondary metabolism may interfere with normal embryogenic development (Gautier et al., 2018; Wang et al., 2022).

Despite these advances, comprehensive metabolomic studies comparing rubber tree genotypes with contrasting embryogenic capacities are lacking. Previous work in *H. brasiliensis* has primarily examined transcriptional changes, with limited attention to metabolic reprogramming and its functional consequences. Because metabolomics captures the end-point of gene expression and protein activity, integrating transcriptomic and metabolomic data can reveal how transcriptional variation translates into metabolic outcomes and how these, in turn, contribute to genotype-dependent SE performance.

In this study, we performed untargeted metabolomic profiling of a high-embryogenic and a low-embryogenic *H. brasiliensis* genotype across SE developmental stages and integrated these data with previously published transcriptomes. Our objectives were to: (1) characterize the metabolic landscape across SE developmental stages; (2) identify DAMs and pathways associated with genotype-dependent embryogenic capacity; (3) elucidate the relationships between secondary metabolite dynamics and SE competence; and (4) identify candidate metabolic and transcriptional regulators that may improve SE efficiency in recalcitrant genotypes. Together, these findings provide new insights into the metabolic determinants of SE competence and offer a theoretical foundation for optimizing SE systems in *H. brasiliensis*.

## 2 Materials and methods

### 2.1 Experimental materials and sample collection

Inflorescences of two *H. brasiliensis* genotypes—Yunyan73477 (high-embryogenic, HE) and Reken628 (low-embryogenic, LE)—were collected during the peak flowering period in March. Immature male flowers (1.3–1.6 mm in diameter) were surface-sterilized with 0.1% HgCl_2_ (BBI Life Sciences, Shanghai, China) for 10 min, followed by thorough rinsing with sterile distilled water. Under aseptic conditions, floral sepals were removed and anthers were excised and placed onto callus induction medium consisting of 1.0 mg L^-1^ 2,4-dichlorophenoxyacetic acid (2,4-D), 1.0 mg L^-1^ kinetin (KT), 1.0 mg L^-1^ naphthaleneacetic acid (NAA), 70 g L^-1^ sucrose, and 50 mL L^-1^ coconut water. Explants were incubated in the dark at 26–28°C for 7–8 weeks to induce loose, pale-yellow callus.

Induced calli were subsequently transferred to differentiation medium containing 1.0 g L^-1^ activated charcoal, 2.0 mg L^-1^ KT, 0.1 mg L^-1^ NAA, 0.5 mg L^-1^ gibberellic acid (GA_3_), 0.2 mg L^-1^ abscisic acid (ABA), 70 g L^-1^ sucrose, and 50 mL L^-1^ coconut water (all reagents from BBI Life Sciences). Cultures were maintained in the dark at 23–25°C. In the HE genotype, globular embryos formed after 3–4 weeks, and cotyledonary embryos developed after 8–9 weeks of continued culture. In contrast, the LE genotype produced only non-embryogenic callus under the same conditions, and no somatic embryos were observed throughout the culture period.

Samples representing different stages of SE were collected for metabolomic and transcriptomic analyses (transcriptome data reported previously in Li et al., 2023). For the HE genotype, four developmental stages were sampled: explants (HE-EX), induced callus (HE-IC), embryogenic callus (HE-EC), and cotyledonary embryos (HE-CE). For the LE genotype, three stages were collected: explants (LE-EX), induced callus (LE-IC), and non-embryogenic callus (LE-C). Three biological replicates were collected for each stage. All samples were immediately frozen in liquid nitrogen and stored at –80°C until further analysis.

### 2.2 Metabolite extraction and UPLC-MS/MS analysis

Freeze-dried samples were prepared using a vacuum freeze dryer (Scientz-100F). The dried tissues were ground into a fine powder using an MM 400 mixer mill (Retsch) with zirconia beads at 30 Hz for 1.5 min. A 50 mg aliquot of powdered tissue was extracted with 1.2 mL of 70% methanol. Samples were vortexed for 30 s every 30 min, for a total of six cycles. After centrifugation at 12,000 rpm for 3 min, the supernatant was filtered through a 0.22 μm membrane (SCAA-104; ANPEL, Shanghai) and used for UPLC-MS/MS analysis.

Metabolomic profiling was performed on a UPLC-ESI-MS/MS system consisting of an ExionLC™ AD UPLC system coupled to an Applied Biosystems 6500 QTRAP mass spectrometer. Chromatographic separation was achieved using an Agilent SB-C18 column (1.8 μm, 2.1 × 100 mm) maintained at 40°C. The mobile phases were: solvent A, water containing 0.1% formic acid; and solvent B, acetonitrile containing 0.1% formic acid. The gradient program was: 0–9 min, 95–5% A; 9–10 min, 5% A; 10–11.1 min, 5–95% A; 11.1–14 min, 95% A. The flow rate was 0.35 mL min^-1^ and the injection volume was 2 μL.

Electrospray ionization (ESI) was conducted in both positive and negative ion modes. Parameters were as follows: ion spray voltage, +5500 V (positive mode) or −4500 V (negative mode); source temperature, 500°C; ion source gas I (GSI), 50 psi; ion source gas II (GSII), 60 psi; curtain gas (CUR), 25 psi; and collision gas set to “high.” Metabolite detection was performed in multiple reaction monitoring (MRM) mode with nitrogen as the collision gas. Declustering potential (DP) and collision energy (CE) were optimized for each MRM transition.

### 2.3 Transcriptome sequencing and data analysis

Transcriptome data for all samples used in this study were obtained from our previously published work (Li et al., 2023). Briefly, RNA extraction and library construction were performed using the same samples, followed by sequencing on the Illumina platform. After quality control of the raw sequencing data, reads were aligned to the rubber tree reference genome (Tang et al., 2016) using HISAT2. DEGs were identified using the DESeq2 package with thresholds of |log_2_FC| ≥ 1 and false discovery rate (FDR) < 0.05. Genes were annotated through BLAST searches against the NR, Swiss-Prot, and KEGG databases. Gene Ontology (GO) and KEGG pathway enrichment analyses were performed using the clusterProfiler package in R.

### 2.4 Metabolomic data processing and statistical analysis

Raw LC-MS/MS data were processed for peak detection, alignment, and quantification using a proprietary data processing pipeline. Metabolites were annotated by comparing retention times, mass spectra, and MS/MS fragmentation patterns with the MetWare Database (MWDB, Metware Biotechnology Co., Ltd., Wuhan, China) and public metabolite libraries.

Principal component analysis (PCA) was performed on the data that were unit variance standardized using the prcomp function in R software. DAMs were identified using orthogonal partial least squares discriminant analysis (OPLS-DA). Variable importance in projection (VIP ≥ 1.0) and fold change (FC ≥ 2 or ≤ 0.5) were used as selection thresholds. OPLS-DA models were constructed after log_2_ transformation and mean centering of metabolite intensities, and model robustness was evaluated by 200 permutation tests (*p* < 0.05).

DAMs were mapped to KEGG pathways using KEGG Compound IDs (Kanehisa and Goto, 2000), and pathway enrichment analysis was conducted using a hypergeometric test with significance set at *p* < 0.05. The same criteria were applied for transcriptome-based pathway enrichment. For multi-omics integration, DEGs and DAMs were jointly mapped to KEGG pathways to identify co-regulated metabolic nodes.

## 3 Results

### 3.1 Metabolite identification and quality control analysis

**Figure 1:**
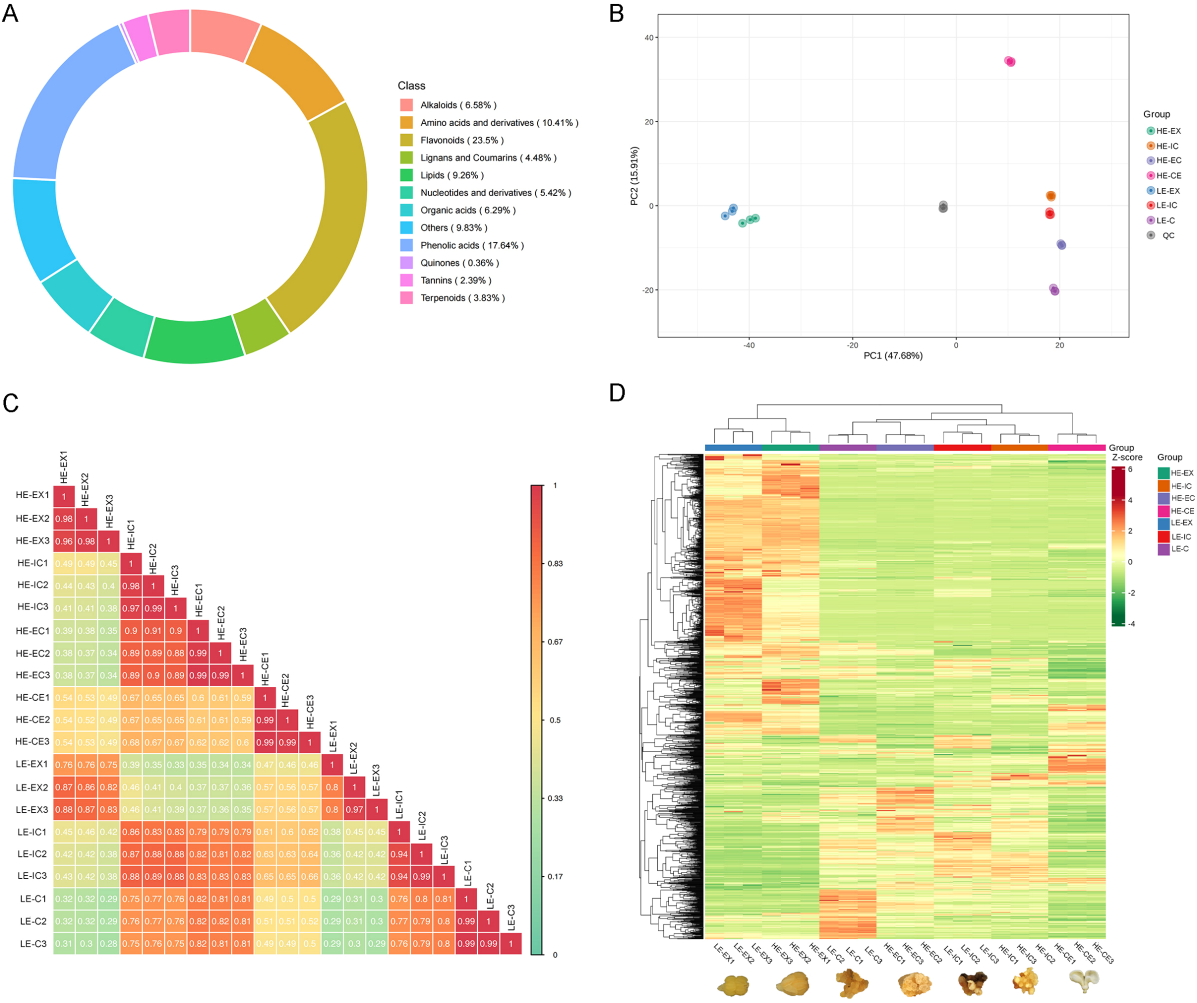
Global metabolomic profiling of rubber tree genotypes with high (HE) and low (LE) embryogenic potential across different developmental stages. **(A)** Donut chart showing the chemical classification of all detected metabolites. **(B)** Principal component analysis score plot of all samples and quality control samples. **(C)** Pearson correlation heatmap among biological replicates. Diagonal and vertical labels indicate sample identities. Colors from blue to red represent strong negative to strong positive correlations, with correlation coefficients shown in each cell. **(D)** Hierarchical clustering heatmap of all metabolites after normalization. Columns represent samples and rows represent metabolites. Colors from green to red indicate low to high relative abundance. The group annotations above the heatmap distinguish different sample categories (HE-EX, HE-IC, HE-EC, HE-CE, LE-EX, LE-IC, LE-C), revealing global metabolic differences and clustering patterns among developmental stages.

Using the UPLC-MS/MS platform combined with the MWDB metabolite database, a total of 1,383 metabolites were identified across all developmental stages of *H. brasiliensis* SE. These metabolites were classified into 11 major categories, with flavonoids accounting for the largest proportion (23.50%), followed by phenolic acids (17.64%) and amino acids and derivatives (10.41%). Other abundant groups included lipids (9.26%), alkaloids (6.58%), organic acids (6.29%), and nucleotides and derivatives (5.42%) (**Figure 1A**).

To ensure data reliability, pooled quality control (QC) samples were included throughout the analytical sequence. The empirical cumulative distribution function (ECDF) analysis showed that the coefficient of variation (CV) distributions of biological replicates from all developmental stages largely overlapped. Across all sample groups, more than 75% of metabolites exhibited CV < 0.3, and over 85% displayed CV < 0.5, indicating excellent instrumental stability and technical reproducibility (**Supplementary Figure S1**). Pearson correlation analysis further confirmed the high consistency among biological replicates (**Figure 1C**). In the HE genotype, correlations ranged from r = 0.96–0.99 across the HE-EX, HE-IC, HE-EC, and HE-CE stages. Similarly, samples from the LE genotype showed correlations of r = 0.94–0.99.

PCA revealed clear metabolic transitions during the SE process. The first two principal components (PC1 and PC2) explained 47.68% and 15.91% of the total variance, respectively, accounting for 63.59% of overall metabolic variation. Samples clustered primarily according to developmental stage rather than genotype, with HE and LE samples at the same stage grouping more closely than samples at different stages within the same genotype (**Figure 1B**). Hierarchical clustering analysis supported this trend, showing tight clustering of biological replicates and stage-dependent separation of samples (**Figure 1D**). Notably, the cotyledonary embryo stage (HE-CE), which is unique to the HE genotype, formed an independent branch in both PCA and hierarchical clustering analyses, indicating distinct metabolic signatures associated with somatic embryo formation.

### 3.2 Identification of DAMs

#### 3.2.1 DAMs between genotypes across developmental stages

**Figure 2:**
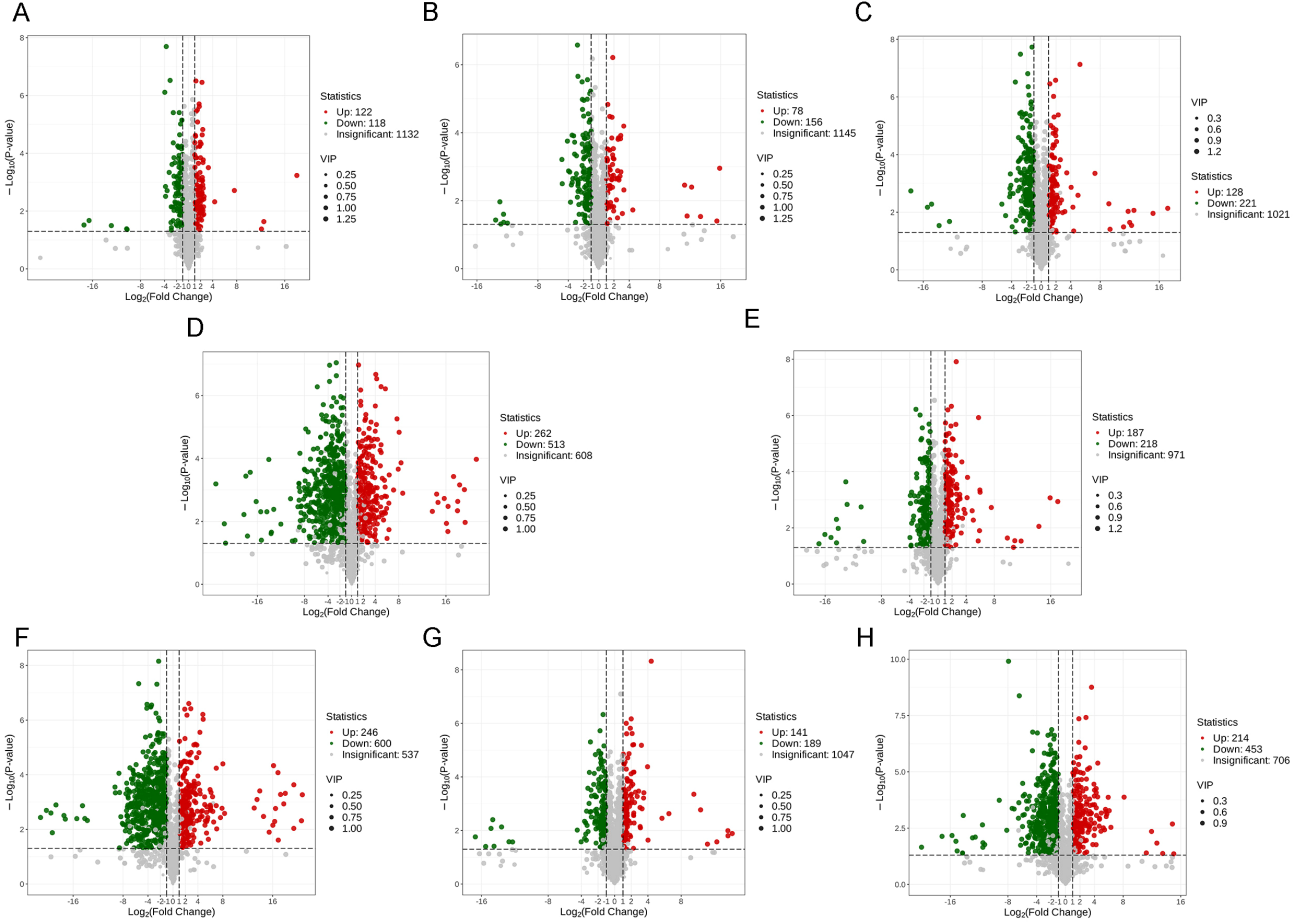
Volcano plots of DAMs across sample comparisons. Volcano plots illustrate the DAMs identified using the triple screening criteria of VIP ≥ 1, fold change ≥ 2 or ≤ 0.5, and *p* < 0.05. Each dot represents a metabolite, with red and green indicating significantly up-regulated and down-regulated metabolites, respectively, and grey representing non-significant metabolites. The x-axis shows the log_2_ fold change (log_2_FC), the y-axis shows the −log_10_*p*-value, and dot size corresponds to the VIP value. **(A–C)** compare the HE and LE genotypes at the same developmental stage: **(A)** LE-EX vs. HE-EX; **(B)** LE-IC vs. HE-IC; **(C)** LE-C vs. HE-EC. **(D, E)** show developmental comparisons within the LE genotype: **(D)** LE-EX vs. LE-IC; **(E)** LE-IC vs. LE-C. Panels F-H show developmental comparisons within the HE genotype: **(F)** HE-EX vs. HE-IC; **(G)** HE-IC vs. HE-EC; **(H)** HE-EC vs. HE-CE.

To investigate metabolic differences underlying embryogenic competence, DAMs were identified using OPLS-DA with thresholds of VIP ≥ 1 and |log_2_FC| ≥ 1 (fold change ≥ 2 or ≤ 0.5). A total of 240, 234, and 349 DAMs were detected during each developmental stage between the two genotypes (LE-EX vs. HE-EX, LE-IC vs. HE-IC, LE-C vs. HE-EC) (**Figures 2A–C**).

**Figure 3:**
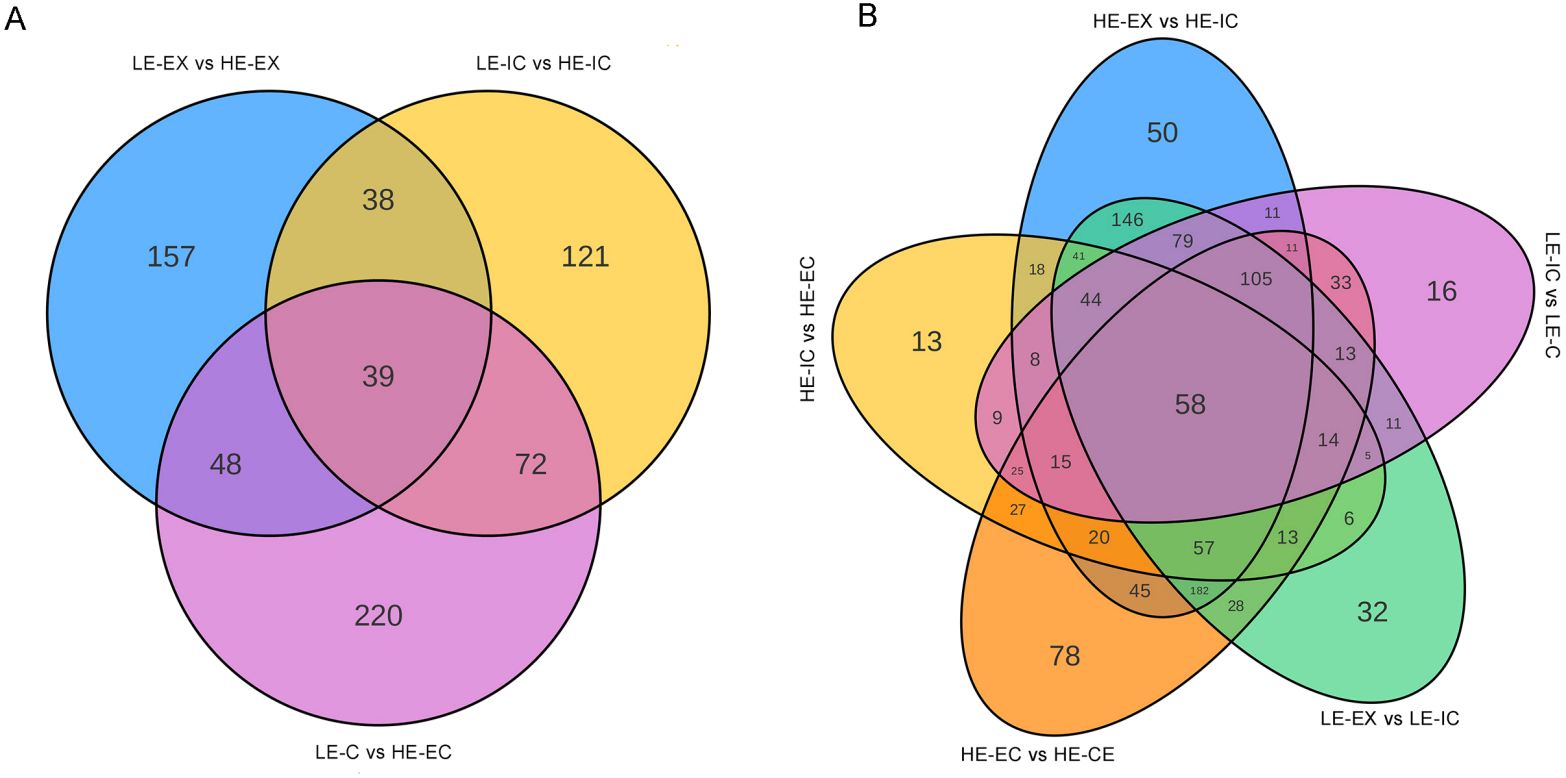
Venn diagrams of DAMs across comparison groups. **(A)** Overlap of DAMs between the two genotypes with contrasting embryogenic potential at the same developmental stage. The comparisons include LE-EX vs. HE-EX, LE-IC vs. HE-IC, and LE-C vs. HE-EC. **(B)** Overlap of DAMs across five developmental comparisons covering both genotypes, including LE-EX vs. LE-IC, LE-IC vs. LE-C, HE-EX vs. HE-IC, HE-IC vs. HE-EC, and HE-EC vs. HE-CE.

Venn diagram analysis showed that the three genotype comparison pairs shared only 39 common DAMs, indicating substantial metabolic divergence across stages. The LE-C vs. HE-EC stage displayed the largest number of stage-specific DAMs (220), which was markedly higher than in the LE-EX vs. HE-EX (157) and LE-IC vs. HE-IC (121) stages. Pairwise comparison further revealed that the LE-IC vs. HE-IC and LE-C vs. HE-EC stages were more similar to each other, sharing 72 DAMs, compared with other stage combinations (**Figure 3A**).

**Figure 4:**
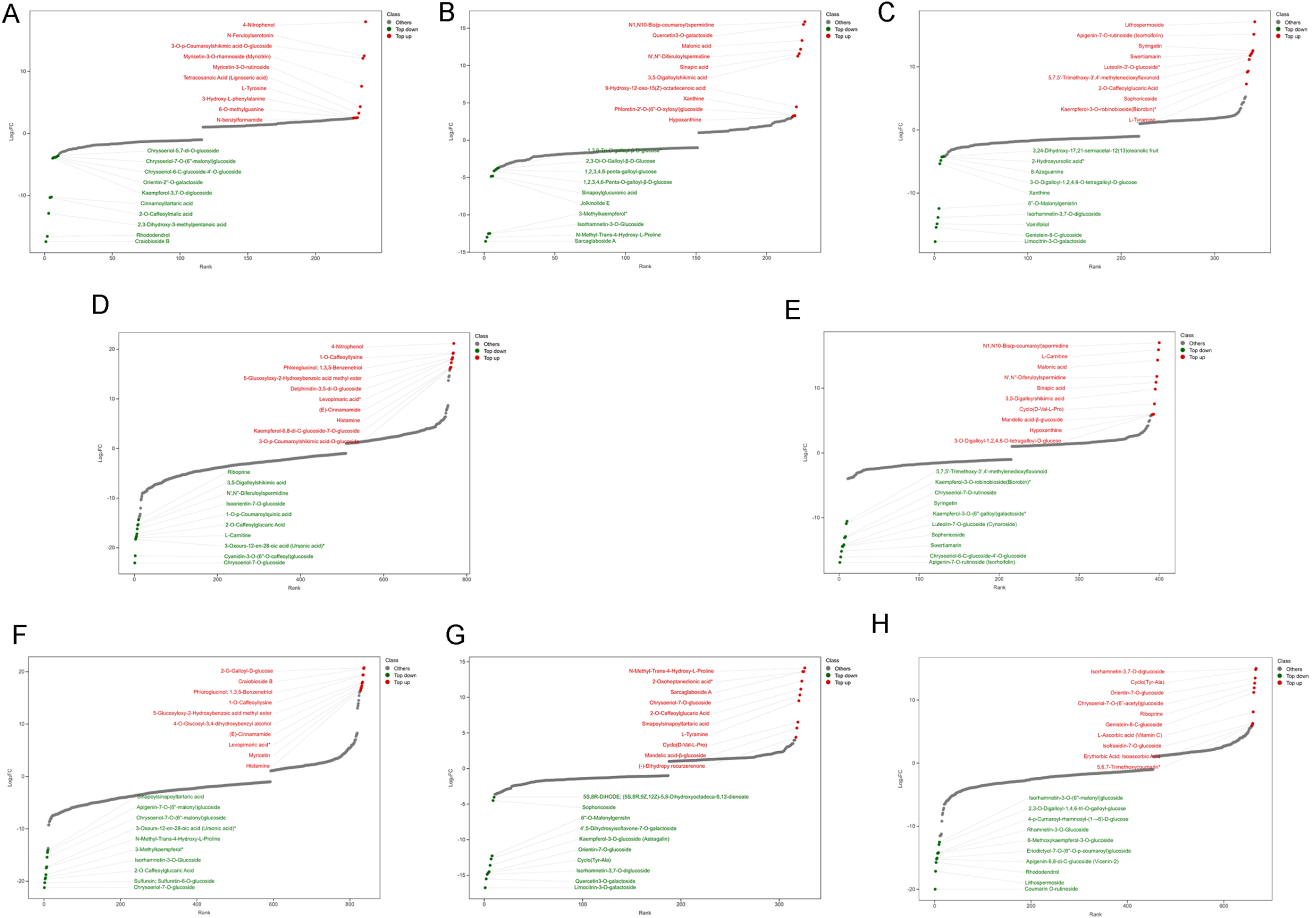
Dynamic distribution of fold-change variations in DAMs across comparison groups. The x-axis represents the cumulative number of metabolites ranked from low to high based on fold change, and the y-axis indicates the log_2_FC. Each dot represents a metabolite, with red and green dots indicating the top 10 most highly up-regulated and down-regulated metabolites, respectively, while grey dots denote other metabolites. **(A–C)** compare the HE and LE genotypes at the same developmental stage: **(A)** LE-EX vs. HE-EX; **(B)** LE-IC vs. HE-IC; **(C)** LE-C vs. HE-EC. **(D–H)** show developmental comparisons within each genotype: **(D)** LE-EX vs. LE-IC; **(E)** LE-IC vs. LE-C; **(F)** HE-EX vs. HE-IC; **(G)** HE-IC vs. HE-EC; **(H)** HE-EC vs. HE-CE.

In LE-EX vs. HE-EX, the top 10 DAMs were mainly enriched in phenolic acids. HE explants showed higher accumulation of N-feruloylserotonin, 3-O-p-coumaroylshikimic acid, and myricetin-3-O-rhamnoside (myricitrin), whereas LE explants accumulated higher levels of rhododendrol, 2,3-dihydroxy-3-methylpentanoic acid, and 2-O-caffeoylmalic acid (**Figure 4A**; **Supplementary Table S1**).

In LE-IC vs. HE-IC, HE callus displayed enriched accumulation of N1,N10-bis(p-coumaroyl)spermidine and quercetin-3-O-galactoside, while the LE genotype accumulated sarcaglaboside A and N-methyl-trans-4-hydroxy-L-proline (**Figure 4B**; **Supplementary Table S1**).

In LE-C vs. HE-EC, the top 10 DAMs were predominantly classified into flavonoids and terpenoids. The LE genotype accumulated high levels of limocitrin-3-O-galactoside, genistein-8-C-glucoside, and vomifoliol, whereas the HE genotype showed significant enrichment of lithospermoside, apigenin-7-O-rutinoside (isorhoifolin), and swertiamarin (**Figure 4C**; **Supplementary Table S1**).

A comparison of DAM categories across stages revealed a transition from phenolic-acid-dominated differences at the explant stage to flavonoids and terpenoids at later stages, reflecting a shift from early stress-responsive metabolism toward specialized metabolic pathways associated with differentiation.

#### 3.2.2 Dynamic metabolic changes during the SE culture process

To characterize stage-specific metabolic reprogramming during SE, metabolites from consecutive developmental stages were compared within each genotype. Substantial metabolic shifts were observed during all developmental stages. A total of 775, 405, 846, 330, and 667 DAMs were identified during the stages LE-EX vs. LE-IC, LE-IC vs. LE-C, HE-EX vs. HE-IC, HE-IC vs. HE-EC, and HE-EC vs. HE-CE, respectively (**Figures 2D–H**).

Venn diagram analysis showed that only 58 DAMs were shared among all five comparisons, confirming that each stage is characterized by a unique metabolic profile. Notably, the extent of metabolic similarity between the two genotypes varied considerably across developmental transitions. During the explant-to-callus transition, 146 DAMs were shared between HE and LE, indicating largely conserved metabolic responses. In contrast, the callus-to-differentiation transition shared only 9 DAMs, reflecting pronounced metabolic divergence at the transition where embryogenic outcomes differ (**Figure 3B**).

##### 3.2.2.1 Metabolic changes during the SE culture process in the LE genotype

In LE-EX vs. LE-IC, several metabolites—including chrysoeriol-7-O-glucoside, cyanidin-3-O-(6’’-O-caffeoyl) glucoside, and 3-oxours-12-en-28-oic acid (ursonic acid)—were significantly downregulated. By contrast, metabolites such as 1-O-caffeoyllysine and phloroglucinol (1,3,5-benzenetriol) displayed strong accumulation (**Figure 4D**; **Supplementary Table S1**).

In LE-IC vs. LE-C, metabolites including N1,N10-bis(p-coumaroyl)spermidine and L-carnitine accumulated strongly, whereas apigenin-7-O-rutinoside (isorhoifolin) and chrysoeriol-6-C-glucoside-4’-O-glucoside were markedly downregulated (**Figure 4E**; **Supplementary Table S1**).

##### 3.2.2.2 Metabolic changes associated with somatic embryogenesis in the HE genotype

In the HE genotype, the HE-EX vs. HE-IC transition featured significant downregulation of several flavonoids, including chrysoeriol-7-O-glucoside and sulfurein (sulfuretin-6-O-glucoside), accompanied by strong accumulation of 2-O-galloyl-D-glucose and craiobioside B (**Figure 4F**; **Supplementary Table S1**).

In HE-IC vs. HE-EC, metabolites such as limocitrin-3-O-galactoside and quercetin-3-O-galactoside were strongly downregulated. In contrast, N-methyl-trans-4-hydroxy-L-proline and 2-oxoheptanedionic acid accumulated substantially (**Figure 4G**; **Supplementary Table S1**).

In HE-EC vs. HE-CE, coumarin O-rutinoside exhibited the strongest decrease. Additionally, metabolites including lithospermoside, rhododendrol, and apigenin-6,8-di-C-glucoside (vicenin-2) were markedly reduced. In contrast, several compounds, such as cyclo (tyr-ala) and L-ascorbic acid (vitamin C), showed strong accumulation (**Figure 4H**; **Supplementary Table S1**).

### 3.3 KEGG pathway enrichment analysis

To further elucidate the metabolic mechanisms underlying the contrasting SE capacities of the two rubber tree genotypes, KEGG enrichment analysis was performed for all DAMs identified across the comparison groups.

**Figure 5:**
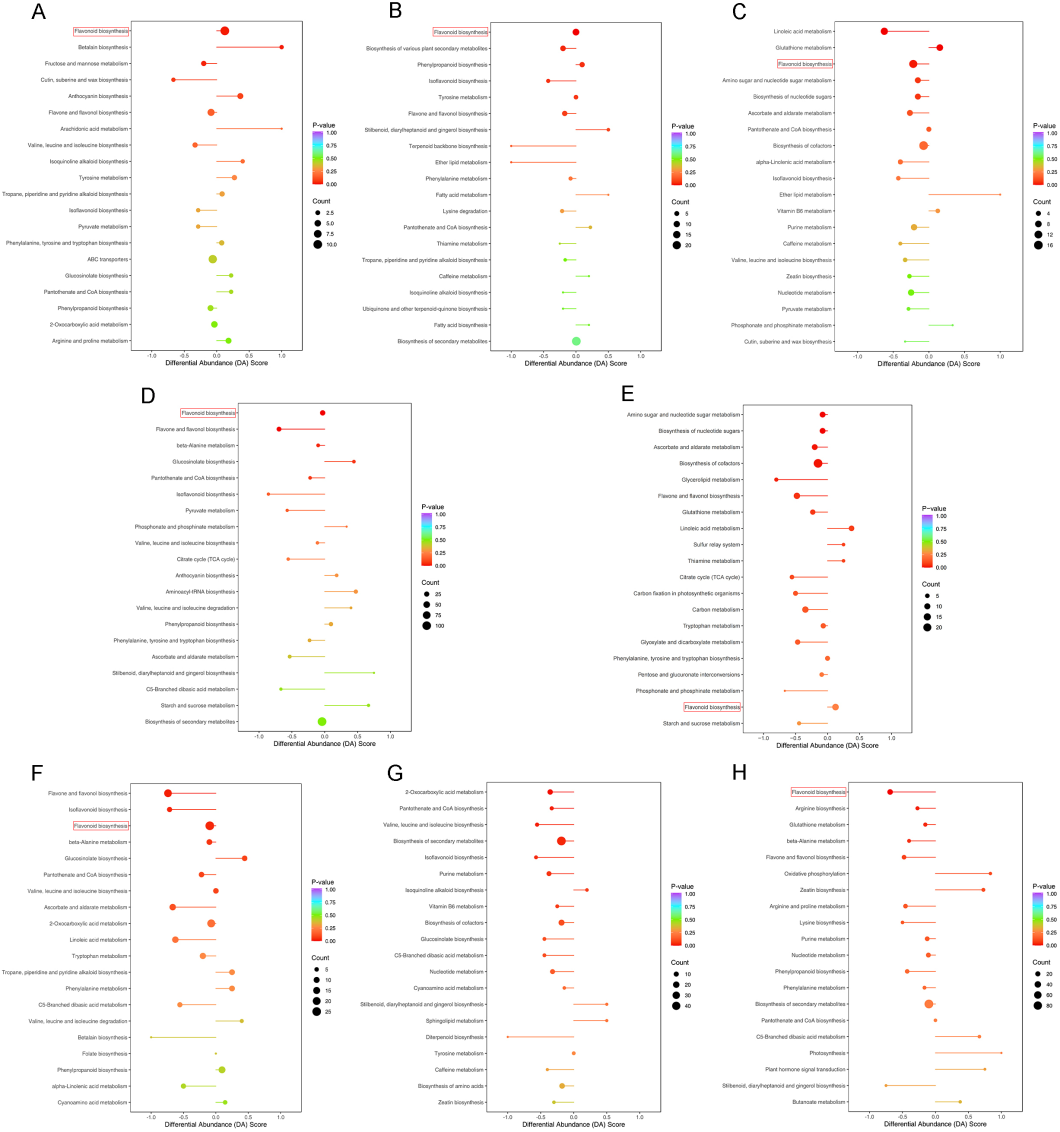
KEGG pathway-level analysis of differential metabolite changes across comparisons. For each comparison, the top 20 KEGG pathways ranked by *p*-value are shown. The y-axis lists pathways ordered by *p*-value, and the x-axis shows the DA Score, which indicates the overall direction of change for all metabolites within each pathway. Bar length reflects the absolute DA Score, dot size indicates the number of differential metabolites, and dot/bar color represents *p*-value (red = lower *p*-value; purple = higher *p*-value). Bars and dots positioned to the right indicate overall up-regulation, whereas those positioned to the left indicate overall down-regulation. Panels correspond to: **(A)** LE-EX vs. HE-EX; **(B)** LE-IC vs. HE-IC; **(C)** LE-C vs. HE-EC; **(D)** LE-EX vs. LE-IC; **(E)** LE-IC vs. LE-C; **(F)** HE-EX vs. HE-IC; **(G)** HE-IC vs. HE-EC; **(H)** HE-EC vs. HE-CE.

Genotype-based comparisons revealed that the flavonoid biosynthesis pathway was significantly enriched (*p* < 0.01) at all developmental stages (LE-EX vs. HE-EX, LE-IC vs. HE-IC, and LE-C vs. HE-EC) (**Figures 5A–C**). This consistent enrichment suggests that flavonoid metabolism is a key distinguishing feature between the two genotypes. Notably, during the LE-C vs. HE-EC stage, linoleic acid metabolism and glutathione metabolism exhibited the strongest enrichment, followed by flavonoid biosynthesis (**Figure 5C**).

Developmental stage-oriented comparisons showed that both genotypes exhibited strong enrichment of flavonoid-related pathways—particularly flavonoid biosynthesis and flavone and flavonol biosynthesis—during the explant-to-callus transition (LE-EX vs. LE-IC; HE-EX vs. HE-IC) (**Figures 5D, F**). In contrast, the subsequent callus-to-differentiation transition (LE-IC vs. LE-C; HE-IC vs. HE-EC) showed pronounced metabolic divergence between the two genotypes (**Figures 5E, G**). The LE genotype displayed significant enrichment in amino sugar and nucleotide sugar metabolism and nucleotide sugars biosynthesis (*p* < 0.01) (**Figure 5E**). Conversely, the HE genotype exhibited strong enrichment in valine, leucine and isoleucine biosynthesis and pantothenate and CoA biosynthesis (**Figure 5G**), pathways essential for amino acid metabolism, lipid metabolism, and cellular energy production.

In HE-EC vs. HE-CE, DAMs associated with the flavonoid biosynthesis pathway were predominantly downregulated (**Figure 5H**; **Supplementary Figure S2**), suggesting that cotyledonary embryo development may involve further suppression of secondary metabolism.

### 3.4 Integrated transcriptome-metabolome analysis of the flavonoid biosynthesis

KEGG enrichment analysis revealed consistent and significant differences in the flavonoid biosynthesis pathway between the two genotypes across multiple developmental stages (**Figure 5**; **Supplementary Figure S3**). To clarify the regulatory roles of this pathway during SE, transcriptomic and metabolomic datasets were integrated, and gene-metabolite co-regulation patterns were compared during two key developmental stages: explant-to-callus induction and callus-to-differentiation (**Supplementary Figure S3**).

#### 3.4.1 Conserved activation of flavonoid biosynthesis during the explant-to-callus transition

**Figure 6:**
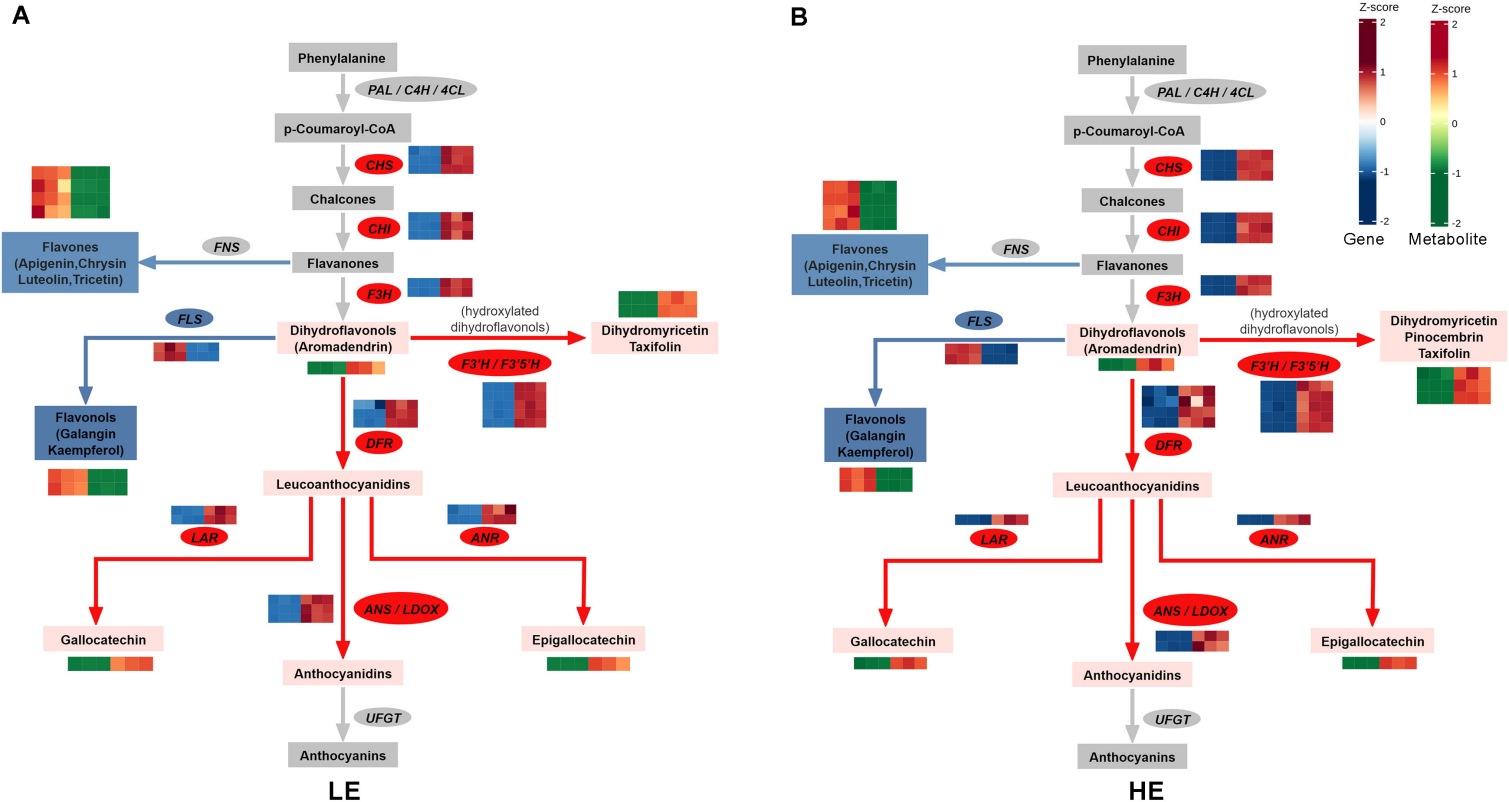
Conserved activation of flavonoid metabolism during the explant-to-callus transition. Integrated transcriptomic and metabolomic analysis of the flavonoid biosynthesis pathway during early dedifferentiation. **(A)** LE genotype (LE-EX vs. LE-IC). **(B)** HE genotype (HE-EX vs. HE-IC). Rectangular boxes represent metabolite pools; ovals represent biosynthetic genes. Color coding indicates overall regulatory direction: red, upregulated/accumulated; blue, downregulated/depleted; gray, not detected or mixed regulation (i.e., multiple gene isoforms showing opposite trends). Heatmaps adjacent to selected genes and metabolites display expression levels or abundance across biological replicates, with the left three cells representing explant stage (EX, three replicates) and the right three cells representing induced callus stage (IC, three replicates). Color intensity represents Z-score normalized expression values for genes (blue, low expression; red, high expression) or relative abundance for metabolites (green, low abundance; red, high abundance).

During the explant-to-callus transition (LE-EX vs. LE-IC; HE-EX vs. HE-IC), the two genotypes exhibited highly similar transcriptional and metabolic responses in the flavonoid biosynthesis pathway (**Figures 6A, B**). In the LE genotype, 101 DEGs and 27 DAMs mapped to this pathway, while the HE genotype contained 95 DEGs and 25 DAMs (**Supplementary Tables S2**-**S5**). Comparative analysis identified 79 co-regulated genes (67.5%) and 24 co-regulated metabolites (85.7%), all showing identical regulatory directions (either both up-regulated or both down-regulated) (**Tables 1**, **2**). These results indicate a conserved regulatory program of flavonoid metabolism during the early dedifferentiation phase.

**Table 1:** DEGs commonly enriched in the flavonoid biosynthesis pathway during the explant-to-callus transition in both genotypes.

Gene ID	NR annotation	Log_2_FC	
		LE-IC vs. LE-C	HE-IC vs. HE-EC
LOC110673296	agmatine coumaroyltransferase-2-like	1.79	3.13
LOC110643471	anthocyanidin 3-O-glucosyltransferase 2-like	-1.34	-2.70
LOC110658732	anthocyanidin 3-O-glucosyltransferase 2-like	2.43	2.81
LOC110658731	anthocyanidin 3-O-glucosyltransferase 2-like isoform X1	2.85	4.09
LOC110658083	anthocyanidin reductase ((2S)-flavan-3-ol-forming)	2.48	2.70
LOC110660191	BAHD acyltransferase At5g47980-like	3.89	2.57
LOC110638079	bifunctional dihydroflavonol 4-reductase/flavanone 4-reductase-like	1.04	1.81
LOC110667357	caffeoyl-CoA O-methyltransferase	-1.77	-3.12
LOC110641698	caffeoyl-CoA O-methyltransferase-like	-1.22	-2.07
LOC110645232	caffeoyl-CoA O-methyltransferase-like	-10.56	-9.43
LOC110641350	chalcone synthase 1	4.69	4.30
LOC110648892	chalcone synthase 2	3.47	3.34
LOC110640364	chalcone synthase 2-like	1.79	3.07
LOC110633200	chalcone synthase-like	-1.61	-1.26
LOC110651405	chalcone–flavonone isomerase 2-like isoform X1	2.42	2.62
LOC110640803	codeine O-demethylase-like	2.67	1.05
LOC110642658	codeine O-demethylase-like	3.85	2.97
LOC110648211	codeine O-demethylase-like	-3.46	-3.85
LOC110668477	cytochrome P450 98A2	-1.28	-2.04
LOC110653493	cytochrome P450 CYP73A100-like	2.93	2.13
LOC110655218	dihydroflavonol 4-reductase-like	4.27	2.86
LOC110655234	dihydroflavonol 4-reductase-like	2.53	1.79
LOC110642702	fatty alcohol:caffeoyl-CoA acyltransferase-like	-3.76	-3.91
LOC110660590	flavonoid 3’,5’-hydroxylase 1-like	6.03	5.20
LOC110663155	flavonoid 3’,5’-hydroxylase 1-like	3.72	2.69
LOC110663157	flavonoid 3’,5’-hydroxylase 1-like	3.74	4.44
LOC110653348	flavonoid 3’,5’-hydroxylase 2-like	6.67	5.50
LOC110636257	flavonoid 3’-monooxygenase	4.06	3.53
LOC110635515	flavonoid 3’-monooxygenase-like	-8.61	-5.14
LOC110655043	flavonol synthase/flavanone 3-hydroxylase	-4.69	-2.96
LOC110665573	flavonol synthase/flavanone 3-hydroxylase-like	-9.69	-12.75
novel.3845	hypothetical protein MANES_07G031500 [Manihot esculenta]	-3.45	-3.87
LOC110640000	leucoanthocyanidin dioxygenase-like	6.52	8.10
LOC110652792	leucoanthocyanidin dioxygenase-like	3.77	2.00
LOC110666021	leucoanthocyanidin reductase-like	2.83	2.53
LOC110652765	licodione synthase-like	-8.80	-8.61
LOC110670394	licodione synthase-like	-4.07	-3.37
LOC110665349	licodione synthase-like, partial	-9.41	-10.57
LOC110642838	naringenin,2-oxoglutarate 3-dioxygenase	1.89	3.02
LOC110641151	naringenin,2-oxoglutarate 3-dioxygenase-like	4.87	4.12
LOC110647757	non-functional NADPH-dependent codeinone reductase 2-like isoform X1	1.16	1.10
LOC110660233	omega-hydroxypalmitate O-feruloyl transferase-like	-1.87	-3.33
LOC110672953	phenolic glucoside malonyltransferase 1-like	-5.28	-2.90
LOC110640236	probable 2-oxoglutarate-dependent dioxygenase At3g111800	-9.06	-10.33
LOC110646442	probable 2-oxoglutarate-dependent dioxygenase At3g111800	-1.37	-4.71
LOC110638682	probable 2-oxoglutarate-dependent dioxygenase At5g05600	-7.77	-10.79
LOC110668057	probable chalcone–flavonone isomerase 3	2.75	3.04
LOC110633904	probable chalcone–flavonone isomerase 3 isoform X1	4.26	4.46
LOC110662985	protein BRI1–5 ENHANCED 1-like	2.07	2.14
LOC110633409	protein ECERIFERUM 26-like	-3.43	-4.59
LOC110653986	protein ECERIFERUM 26-like	-8.58	-9.27
LOC110654081	protein ECERIFERUM 26-like	-11.72	-11.12
LOC110637122	protein ECERIFERUM 2-like	-1.15	-4.68
LOC110649734	protein ECERIFERUM 2-like isoform X1	-11.08	-8.35
LOC110640804	protein SRG1-like	1.75	1.03
LOC110643140	protein SRG1-like	-1.22	-1.64
LOC110644267	protein SRG1-like	-2.63	-7.48
LOC110658672	protein SRG1-like	2.79	1.55
LOC110651006	protein SRG1-like [Manihot esculenta]	-2.63	-5.11
LOC110664234	protein SRG1-like isoform X2	1.06	2.94
LOC110650078	shikimate O-hydroxycinnamoyltransferase-like	-1.31	-1.16
LOC110637148	trans-cinnamate 4-monooxygenase	-1.58	-1.66
LOC110665143	trans-cinnamate 4-monooxygenase-like	-3.31	-2.54
LOC110666993	type III polyketide synthase A-like	-12.23	-9.51
LOC110668970	type III polyketide synthase B	-8.92	-6.06
LOC110654731	UDP-glycosyltransferase 43-like	-2.32	-2.71
LOC110666367	UDP-glycosyltransferase 71K1-like isoform X1	-1.22	-1.37
LOC110649415	UDP-glycosyltransferase 71K1-like isoform X2	-2.80	-2.14
LOC110658208	UDP-glycosyltransferase 88A1-like	1.87	2.07
LOC110670575	UDP-glycosyltransferase 88A1-like	-7.77	-4.99
LOC110670671	UDP-glycosyltransferase 88A1-like	-6.68	-6.48
LOC110658204	UDP-glycosyltransferase 88A1-like isoform X1	1.98	2.07
LOC110642627	UDP-glycosyltransferase 88F4-like	-9.15	-9.47
LOC110656234	vestitone reductase-like isoform X1	-5.46	-8.68
LOC110662998	vestitone reductase-like isoform X1	-4.15	-4.19
LOC110636943	vinorine synthase-like	-10.27	-9.94
LOC110660992	vinorine synthase-like	-2.35	-5.59
LOC110667245	vinorine synthase-like	-2.90	-1.65
LOC110667252	vinorine synthase-like	-6.61	-1.95

This table lists the 79 DEGs (*p* < 0.05) that were commonly enriched in the flavonoid biosynthesis pathway in both LE-EX vs. LE-IC and HE-EX vs. HE-IC comparisons.

**Table 2:** DAMs commonly enriched in the flavonoid biosynthesis pathway during the explant-to-callus transition in both genotypes.

Metabolite ID	Compounds	LE-IC vs. LE-C		HE-IC vs. HE-EC	
		Log_2_FC	VIP	Log_2_FC	VIP
Hmln002806	5-O-Caffeoylshikimic acid	3.99	1.05	5.58	1.08
pmb3074	5-O-p-Coumaroylquinic acid	2.84	1.11	2.94	1.09
pmp000571	Apigenin	-6.20	1.11	-6.67	1.09
mws0048	Apigenin-8-C-Glucoside (Vitexin)	-1.15	1.08	-3.04	1.08
mws1094	Aromadendrin (Dihydrokaempferol)	1.34	1.10	1.79	1.08
pme3475	Butin; 7,3’,4’-Trihydroxyflavanone	-3.99	1.11	-4.08	1.09
mws0040	Chrysin	-2.91	1.09	-4.80	1.06
MWSHY0048	Dihydromyricetin (Ampelopsin)	5.67	1.10	7.87	1.06
mws0042	Epigallocatechin	4.03	1.11	3.42	1.09
Lmyn006227	Galangin (3,5,7-Trihydroxyflavone)	-5.91	1.11	-6.76	1.09
mws0049	Gallocatechin	3.12	1.11	2.66	1.08
Lmzp002365	Hesperetin-7-O-glucoside	-3.28	1.10	-2.69	1.09
mws1068	Kaempferol (3,5,7,4’-Tetrahydroxyflavone)	-3.88	1.10	-4.38	1.08
pme0088	Luteolin (5,7,3’,4’-Tetrahydroxyflavone)	-7.17	1.10	-7.21	1.08
mws0032	Myricetin	4.31	1.05	16.84	1.09
MWSHY0137	Naringenin (5,7,4’-Trihydroxyflavanone)	-3.53	1.05	-4.04	1.09
pme2960	Naringenin chalcone; 2’,4,4’,6’-Tetrahydroxychalcone	-3.39	1.09	-4.08	1.09
mws1179	Naringenin-7-O-glucoside (Prunin)	-2.73	1.11	-2.91	1.08
mws0046	Naringenin-7-O-Neohesperidoside(Naringin)	-3.87	1.09	-3.15	1.08
mws2118	Phloretin-2’-O-glucoside (Phlorizin)	2.60	1.08	1.25	1.06
mws0789	Pinocembrin (Dihydrochrysin)	2.95	1.10	2.31	1.08
mws0044	Taxifolin(Dihydroquercetin)	4.83	1.11	5.95	1.09
pmb0751	Trans-5-O-(p-Coumaroyl)shikimate	3.42	1.07	3.92	1.06
mws0920	Tricetin (5,7,3’,4’,5’-Pentahydroxyflavone)	-1.28	1.09	-1.91	1.08

This table lists the 24 DAMs (*p* < 0.05) that were commonly enriched in the flavonoid biosynthesis pathway in both the LE-EX vs. LE-IC and HE-EX vs. HE-IC comparisons.

Integration of transcriptomic and metabolomic data revealed strong activation of the anthocyanin precursor branch in both genotypes. Upstream structural genes—including members of the *chalcone synthase* (*CHS*; LOC110641350, LOC110648892, LOC110640364) and *chalcone-flavanone isomerase(CHI*; LOC110651405, LOC110668057) families—were consistently upregulated, indicating increased flux from the phenylpropanoid pathway into flavonoid biosynthesis (**Figure 6**; **Table 1**).

Downstream regulatory genes showed similar activation patterns. Key enzymes, such as *flavanone 3-hydroxylase* (*F3H*; LOC110641151, LOC110642838), *dihydroflavonol 4-reductase* (*DFR*; LOC110655218, LOC110655234), and *leucoanthocyanidin dioxygenase*/*anthocyanidin synthase* (*LDOX*/*ANS*; LOC110652792, LOC110640000), were significantly upregulated, supporting elevated production of anthocyanin precursors. Correspondingly, several flavanone intermediates, including naringenin and butin, showed pronounced depletion, consistent with their rapid conversion into downstream products under enhanced metabolic flux. (**Figures 6**; **Supplementary Figure S4**; **Tables 1**, **2**).

Notably, multiple *flavonoid 3’,5’-hydroxylase* (*F3’5’H*) genes (LOC110660590, LOC110653348, LOC110663155, LOC110663157) and the *flavonoid 3’-monooxygenase* (*F3’H*) gene (LOC110636257) exhibited strong induction. Consistent with this, dihydromyricetin (ampelopsin) showed the highest accumulation (log_2_FC = 5.67 (LE) and 7.87 (HE)), followed by taxifolin (dihydroquercetin) (log_2_FC = 4.83 (LE) and 5.95 (HE)) and pinocembrin (dihydrochrysin) (log_2_FC = 1.34 (LE) and 1.79 (HE)) (**Figures 6**; **Tables 1**, **2**). Because highly hydroxylated flavonoids possess strong antioxidant activity. These results indicate that both genotypes favored the accumulation of highly hydroxylated flavonoids with strong antioxidant potential during stress-associated dedifferentiation.

Genes involved in the reductive branch of anthocyanin metabolism also showed coordinated induction. *Anthocyanidin reductase* (*ANR*; LOC110658083) and *leucoanthocyanidin reductase* (*LAR*; LOC110666021) were upregulated in both genotypes. Correspondingly, the proanthocyanidin monomers epigallocatechin and gallocatechin accumulated significantly (**Figures 6**; **Tables 1**, **2**). These findings confirm a coordinated activation of the anthocyanin precursor branch in both genotypes.

In stark contrast to the strong activation of the anthocyanin precursor pathway, the flavonol synthesis pathway was consistently suppressed in both genotypes. *Flavonol synthase* (*FLS*) genes LOC110665573 and LOC110655043 exhibited strong downregulation (**Figure 6**; **Supplementary Figure S4**). *FLS* catalyzes the conversion of dihydroflavonols to flavonols, and its downregulation led to a shift in carbon flow from flavonol synthesis to anthocyanin and proanthocyanidin synthesis. Consistent with this transcriptional suppression, flavonol metabolites such as galangin (3,5,7-trihydroxyflavone) were significantly reduced. Several flavone compounds, including apigenin and luteolin (5,7,3’,4’-tetrahydroxyflavone), decreased, while the flavanone pinocembrin pinocembrin increased (**Figure 6**; **Table 2**). Together, these changes demonstrate a conserved metabolic reallocation away from the flavonol branch during early dedifferentiation.

#### 3.4.2 Metabolic differentiation in the flavonoid biosynthesis pathway during the callus-to-differentiation transition

**Figure 7:**
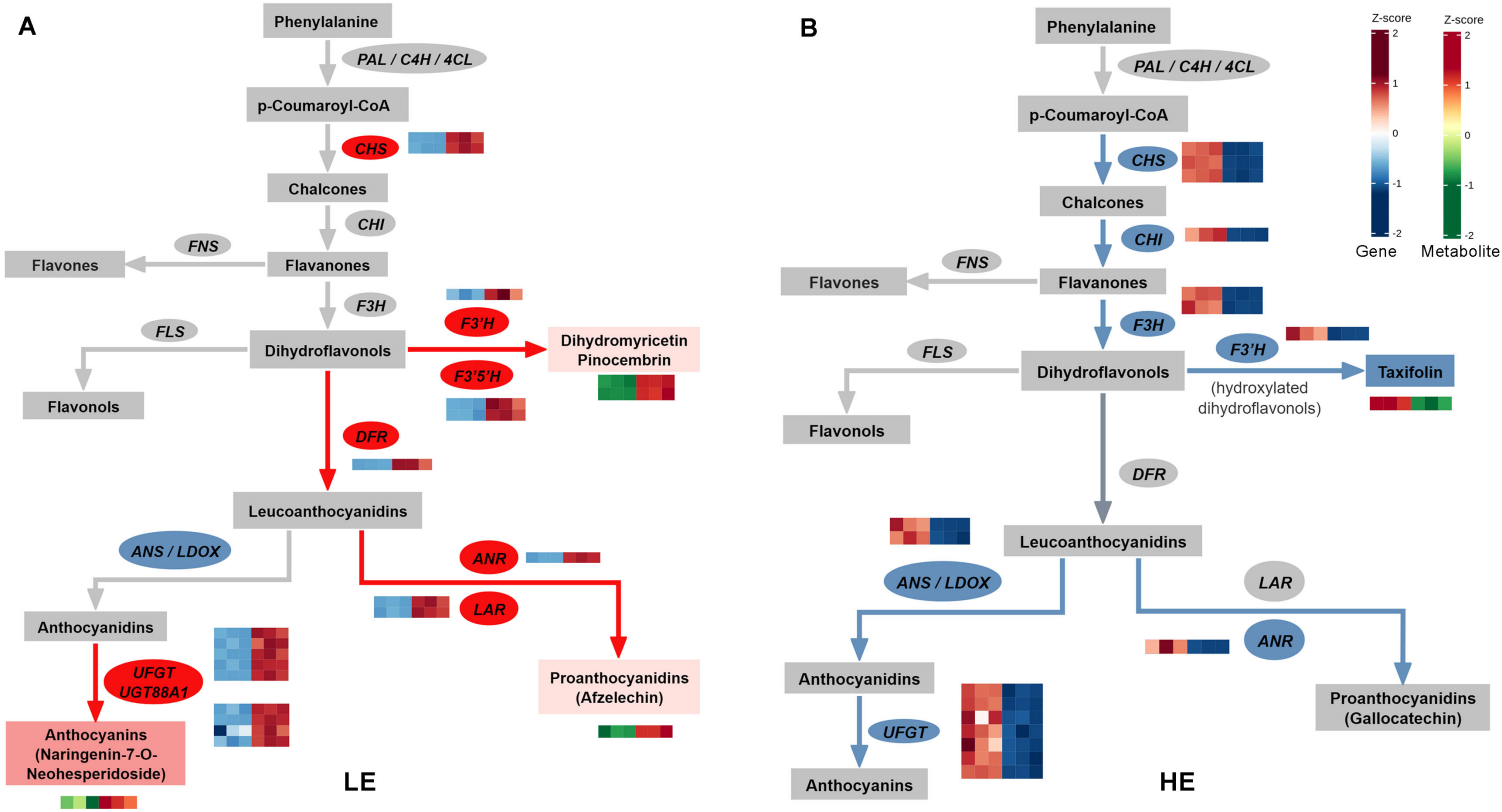
Divergent metabolic regulation in the flavonoid biosynthesis pathway during the callus-to-differentiation transition. Integrated transcriptomic and metabolomic analysis of the flavonoid biosynthesis pathway during somatic embryo differentiation showing contrasting regulatory strategies between genotypes. **(A)** LE genotype (LE-IC vs. LE-C) exhibiting maintenance-type metabolism. **(B)** HE genotype (HE-IC vs. HE-EC) exhibiting conversion-type metabolism. Rectangular boxes represent metabolite pools; ovals represent biosynthetic genes. Color coding indicates overall regulatory direction: red, upregulated/accumulated; blue, downregulated/depleted; gray, not detected or mixed regulation. Heatmaps adjacent to selected genes and metabolites display expression levels or abundance across biological replicates, with the left three cells representing induced callus stage (IC, three replicates) and the right three cells representing differentiation stage (C or EC, three replicates). Color intensity represents Z-score normalized expression values for genes (blue, low expression; red, high expression) or relative abundance for metabolites (green, low abundance; red, high abundance). Red arrows indicate active metabolic flux; blue arrows indicate suppressed flux.

**Figure 8:**
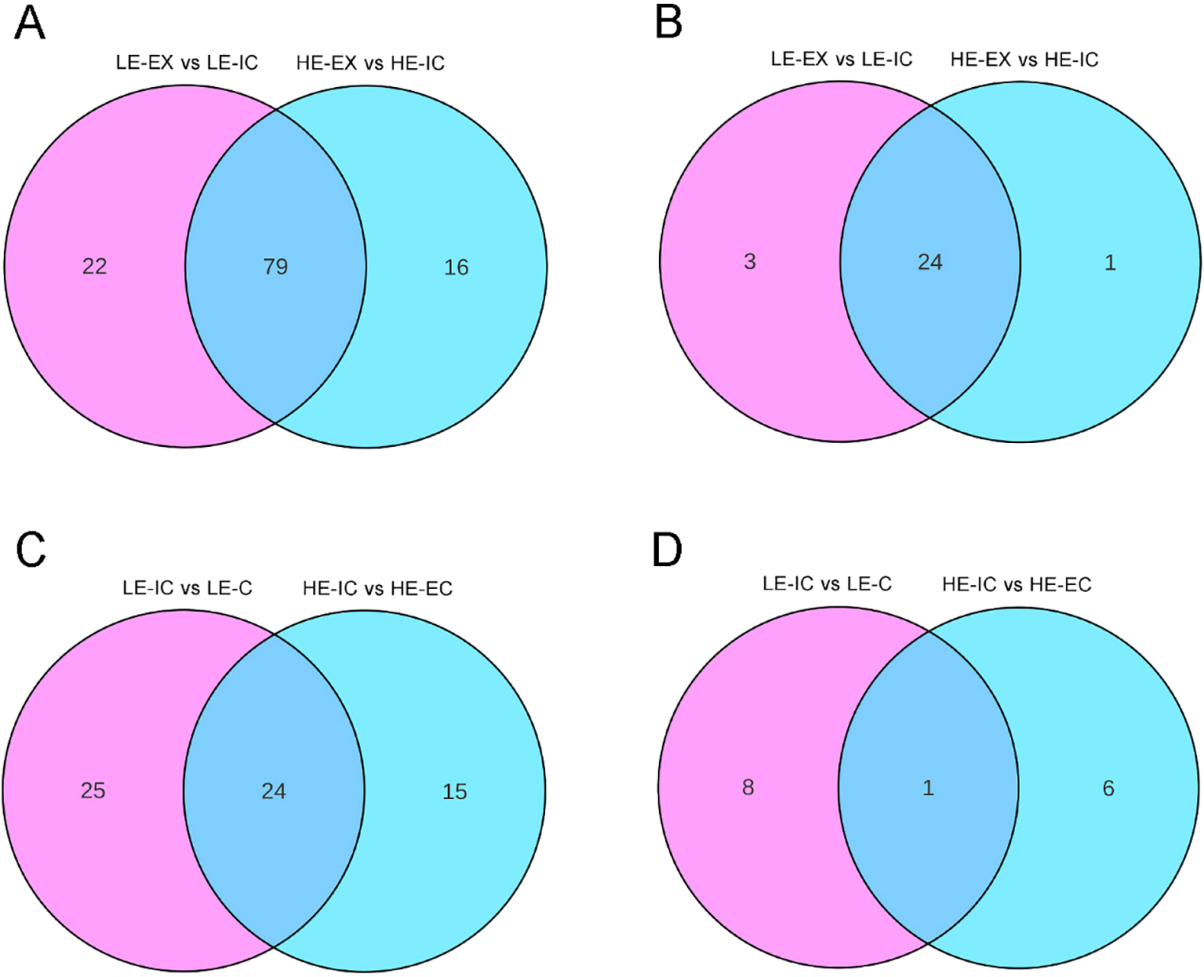
Venn diagrams of DEGs and DAMs enriched in the flavonoid biosynthesis pathway during the explant-to-callus transition and callus-to-differentiation transitions in the two genotypes. **(A)** Venn diagram showing the overlap of DEGs enriched in the flavonoid biosynthesis pathway between LE-EX vs. LE-IC and HE-EX vs. HE-IC during the explant-to-callus transition. **(B)** Venn diagram showing the overlap of DAMs enriched in the flavonoid biosynthesis pathway between LE-EX vs. LE-IC and HE-EX vs. HE-IC during the explant-to-callus transition. **(C)** Venn diagram illustrating the shared DEGs enriched in the flavonoid biosynthesis pathway between LE-IC vs. LE-C and HE-IC vs. HE-EC during the callus-to-differentiation transition. **(D)** Venn diagram illustrating the shared DAMs enriched in the flavonoid biosynthesis pathway between LE-IC vs. LE-C and HE-IC vs. HE-EC during the callus-to-differentiation transition.

Despite the highly conserved metabolic responses observed during the explant-to-callus transition, the two genotypes exhibited strikingly divergent regulatory patterns during the callus-to-differentiation transition (LE-IC vs. LE-C; HE-IC vs. HE-EC) (**Figures 7**). The LE genotype contained 49 DEGs and 9 DAMs mapped to the flavonoid pathway, whereas the HE genotype contained 39 DEGs and 7 DAMs (**Supplementary Tables S3**-**S9**). Only 24 DEGs (37.5%) and one metabolite (6.7%) were co-regulated between genotypes, confirming dramatic pathway divergence (**Figures 8C, D**). Importantly, 11 key genes exhibited completely opposite regulatory directions between the two genotypes (**Table 3**). These included two *CHS* genes and five *anthocyanidin 3-O-glucosyltransferase 2-like genes* (*UFGT*), and several additional genes involved in modification and regulatory processes (**Table 3**).

**Table 3:** DEGs commonly enriched in the flavonoid biosynthesis pathway during the callus-to-differentiation transition in two genotypes.

Gene ID	NR annotation	Log_2_FC	
		LE-IC vs. LE-C	HE-IC vs. HE-EC
LOC110673296	agmatine coumaroyltransferase-2-like	1.62	1.92
LOC110658723	anthocyanidin 3-O-glucosyltransferase 1-like	-1.29	-3.05
LOC110658726	anthocyanidin 3-O-glucosyltransferase 1-like	-1.36	-2.22
LOC110642702	fatty alcohol:caffeoyl-CoA acyltransferase-like	-7.90	-2.21
LOC110640000	leucoanthocyanidin dioxygenase-like	-2.47	-4.13
LOC110660233	omega-hydroxypalmitate O-feruloyl transferase-like	2.48	3.28
LOC110633683	probable caffeoyl-CoA O-methyltransferase At4g26220	1.85	1.24
LOC110633409	protein ECERIFERUM 26-like	1.14	1.98
novel.2231	protein SRG1-like	2.34	1.21
LOC110651006	protein SRG1-like [Manihot esculenta]	3.29	2.25
LOC110650078	shikimate O-hydroxycinnamoyltransferase-like	2.72	1.51
novel.2753	UDP-glycosyltransferase 88B1 [Jatropha curcas]	-4.13	-4.21
LOC110660132	vinorine synthase-like	2.95	4.45
LOC110643466	anthocyanidin 3-O-glucosyltransferase 2-like	1.77	-1.63
LOC110649753	anthocyanidin 3-O-glucosyltransferase 2-like	1.18	-2.71
LOC110658732	anthocyanidin 3-O-glucosyltransferase 2-like	1.41	-1.30
novel.2681	anthocyanidin 3-O-glucosyltransferase 2-like	1.04	-2.32
novel.3247	anthocyanidin 3-O-glucosyltransferase 2-like	1.00	-2.38
LOC110648892	chalcone synthase 2	1.08	-1.20
LOC110640364	chalcone synthase 2-like	1.70	-1.40
LOC110670394	licodione synthase-like	-1.64	1.02
LOC110632801	omega-hydroxypalmitate O-feruloyl transferase-like	1.40	-1.36
LOC110668584	protein SRG1-like	1.42	-1.13
LOC110658208	UDP-glycosyltransferase 88A1-like	1.22	-1.38

This table lists the 24 DEGs (*p* < 0.05) that were commonly enriched in the flavonoid biosynthesis pathway in both the LE-IC vs. LE-C and HE-IC vs. HE-EC comparisons. Among these genes, the last 11 exhibit opposite expression trends between the two genotypes.

##### 3.4.2.1 Metabolic features of the flavonoid biosynthesis pathway in the LE genotype

The LE genotype exhibited a maintenance-type metabolic pattern during the LE-IC vs. LE-C transition, characterized by sustained secondary metabolic activity. Although the expression of *CHS* genes decreased during the LE-IC vs. LE-C transition compared with the preceding LE-EX vs. LE-IC transition (log_2_FC reduced from 3.47/1.79 to 1.08/1.70), their expression levels remained above baseline (**Figures 6A**, **7A**). DFR showed a similar reduction (log_2_FC from 2.53 to 1.23; **Supplementary Tables S2**, **S6**; **Figures 6A**, **7A**). Yet dihydroflavonols such as dihydromyricetin continued to accumulate (log_2_FC decreased from 5.67 to 2.77; **Supplementary Tables S4**, **S8**; **Figure 6A**; **Figure 7A**). These results indicate partial attenuation rather than shutdown of flavonoid precursor biosynthesis.

In contrast, the glycosylation machinery became further activated. Five *UFGT* genes were upregulated (**Figure 7A**; **Supplementary Figure S5**). Additionally, *UDP-glycosyltransferase 88A1-like* (LOC110670639) switched from downregulation during the LE-EX vs. LE-IC transition to upregulation during LE-IC vs. LE-C transition (log_2_FC from –1.16 to 2.13). Although the expression of the *SRG1-like* gene (LOC110668584) declined, it remained upregulated overall (log_2_FC from 4.01 to 1.42; **Supplementary Tables S2**, **S6**). Glycosylation reactions require substantial amounts of UDP-glucose, which is consistent with the significant enrichment of the amino sugar and nucleotide sugar metabolism pathway observed in the LE genotype during this transition (**Figure 5E**).

The LE genotype also exhibited a reallocation of upstream carbon flux during the LE-IC vs. LE-C transition. Both *cinnamoyl-CoA reductase* (*CCR*) (novel.160) and *shikimate O-hydroxycinnamoyltransferase* (*HCT*) (LOC110650078) were markedly upregulated, accompanied by continued accumulation of 5-O-caffeoylshikimic acid (**Supplementary Tables S6**, **S8**). In addition, the *omega-hydroxypalmitate O-feruloyl transferase-like* gene (LOC110632801) transitioned from downregulation in the LE-EX vs. LE-IC transition (log_2_FC = –2.64) to upregulation during LE-IC vs. LE-C (log_2_FC = 1.40). Conversely, the *licodione synthase-like* gene (LOC110670394), which was strongly downregulated during the LE-EX vs. LE-IC transition (log_2_FC = –4.07), remained repressed during LE-IC vs. LE-C transition (log_2_FC = –1.64; **Supplementary Tables S2**, **S6**). Correspondingly, glycosylated flavonoids such as naringenin-7-O-neohesperidoside (naringin) showed increased accumulation (log_2_FC = 1.31), confirming enhanced glycosylation activity (**Supplementary Tables S8**).

Together, these results demonstrate that the LE genotype maintains persistent secondary metabolic activity during the LE-IC vs. LE-C transition. Although the synthesis rate of anthocyanin precursors diminishes, glycosylation processes are further enhanced and a portion of the carbon flux is redirected toward phenolic acid biosynthesis and fatty acid modification.

##### 3.4.2.2 Metabolic features of the flavonoid biosynthesis pathway in the HE genotype

In sharp contrast to the LE genotype, the HE genotype exhibited a conversion-type metabolic pattern, characterized by a coordinated suppression of the core flavonoid biosynthetic machinery, accompanied by residual accumulation of selected intermediates during the HE-IC vs. HE-EC transition. Downstream dihydroflavonols such as taxifolin also declined (log_2_FC = -1.15), contrasting with their sustained accumulation in the LE genotype. *CHS* genes were markedly downregulated (**Figure 7B**; **Table 3**), showing an opposite regulatory trend compared with the sustained upregulation observed in the LE genotype. Consistently, the downstream genes *CHI* and *F3H* also showed significant repression. As *CHS* serves as a major rate-limiting enzyme in the pathway, its suppression effectively reduced carbon flux entering flavonoid biosynthesis. This shift likely enabled the HE genotype to redirect carbon resources toward primary metabolism—particularly amino acid and energy metabolism, as indicated by KEGG enrichment (**Figure 5G**).

The glycosylation machinery of the HE genotype was similarly repressed during the HE-IC vs. HE-EC transition. All five *UFGT* genes were downregulated (**Figure 7B**; **Table 3**). In addition, two anthocyanidin 3-O-glucosyltransferase 1-like genes showed stronger repression in HE (log_2_FC –2.22 and –3.05) compared with LE (–1.36 and –1.29) (**Table 3**). The downstream anthocyanin biosynthesis branch also experienced intensified repression. *LDOX* displayed substantial downregulation, and the HE genotype further repressed *ANR*, a gene that showed up-regulated in the LE genotype (**Figure 7**). These results indicate that both the biosynthesis and modification of anthocyanins were strongly inhibited during somatic embryo differentiation in the HE genotype.

Collectively, the HE genotype exhibits a metabolic reprogramming strategy during the HE-IC vs. HE-EC transition. From the suppression of the upstream rate-limiting enzyme *CHS* to the coordinated downregulation of the glycosylation system, the flavonoid biosynthetic pathway is repressed at both transcriptional and metabolic levels. Such a shift likely reallocating carbon, sugar donors (e.g., UDP-glucose), and energy toward primary metabolic pathways essential for embryogenic transition. This interpretation is supported by the significant enrichment of amino acid biosynthesis and CoA-related metabolic pathways in the HE genotype (**Figure 5G**), suggesting a strategic shift from secondary to primary metabolism.

## 4 Discussion

### 4.1 Metabolic reprogramming as a central feature of SE

By integrating transcriptomic and metabolomic datasets, this study provides a comprehensive view of the metabolic dynamics underlying SE in *H. brasiliensis*, and highlights distinct metabolic behaviors between high- and low-embryogenic genotypes across developmental transitions. A total of 1,383 metabolites were identified, spanning 11 major metabolic categories. Among these, flavonoids, phenolic acids, and amino acids and their derivatives represented the dominant metabolite classes. PCA revealed that samples clustered primarily according to developmental transition rather than genotype, with the first two components explaining 63.59% of the total variance. This pattern indicates that developmental progression is the major driver of metabolic shifts during SE.

Among genotype-based comparisons, 240, 234, and 349 DAMs were detected at the LE-EX vs. HE-EX, LE-IC vs. HE-IC, and LE-C vs. HE-EC transitions, respectively, with the LE-C vs. HE-EC transition showing the highest metabolic divergence. More importantly, the functional categories of these metabolic differences underwent a marked transition. Early SE stages were dominated by secondary metabolic pathways, such as flavonoid and phenylpropanoid biosynthesis, whereas the LE-C vs. HE-EC transition was characterized by the activation of primary metabolic pathways, including linoleic acid metabolism, glutathione metabolism, and amino sugar and nucleotide sugar metabolism. This shift from secondary to primary metabolic dominance reflects a profound metabolic reprogramming process, which appears essential for successful SE progression.

Such metabolic reprogramming is consistent with observations from several other plant species. In multiple SE processes, early dedifferentiation is often associated with stress-related pathways (Fehér, 2015; Nic-Can and Loyola-Vargas, 2016; Ge et al., 2025). In *Gossypium hirsutum*, for example, high-embryogenic genotypes show DEGs enriched in fatty acid, tryptophan, and pyruvate metabolism, whereas low-embryogenic genotypes exhibit enrichment in pathways related to DNA conformational changes, resulting in divergent metabolic trajectories (Guo et al., 2020). A similar pattern has been reported in *Coffea arabica*, where SE involves a coordinated transition between stress-associated metabolism and growth-related primary metabolic pathways (Awada et al., 2019, Awada et al., 2023).

### 4.2 Conserved activation of flavonoid biosynthesis during callus induction

KEGG enrichment analysis revealed that the flavonoid biosynthesis pathway was significantly enriched in both developmental and genotype-based comparisons. Integrated transcriptome-metabolome analysis further demonstrated that this pathway is tightly co-regulated during early dedifferentiation, followed by pronounced divergence during the callus-to-differentiation transition. During the explant-to-callus transition, both genotypes exhibited highly consistent transcriptional and metabolic responses in the flavonoid biosynthesis pathway, with 67.5% of DEGs and 85.7% of DAMs shared between genotypes, all showing identical regulatory directions. In contrast, during the callus-to-differentiation transition, only 37.5% of DEGs and 6.7% of DAMs were shared, and 11 key genes displayed entirely opposite regulatory trends.

The early-stage conservation was primarily reflected in the coordinated activation of the anthocyanin precursor biosynthetic branch. From *CHS* (carbon backbone synthesis) through *CHI*, *F3H*, *DFR*, and *LDOX* (progressive structural modifications), multiple core enzymes were significantly upregulated in both genotypes. This activation was accompanied by pronounced depletion of flavanone precursors (naringenin and butin), together with strong accumulation of downstream dihydroflavonols (aromadendrin, taxifolin, and dihydromyricetin), consistent with enhanced metabolic flux through the anthocyanin precursor branch. Meanwhile, *FLS* genes were strongly downregulated (log_2_FC –9.69 to –12.75), together with a marked reduction in flavonol metabolites such as galangin and quercetin. Flavone compounds (apigenin, luteolin) also decreased. This metabolic partitioning suggests that carbon flux during callus formation is preferentially directed toward anthocyanin and proanthocyanidin biosynthesis rather than flavonol production. Consistent with this trend, *ANR* and *LAR* showed coordinated upregulation, and the corresponding proanthocyanidin monomers epigallocatechin and gallocatechin accumulated robustly.

The conserved activation of the flavonoid pathway during early dedifferentiation likely reflects a shared stress response triggered by tissue culture conditions. Explant excision and callus induction generate substantial oxidative stress, necessitating enhanced antioxidant defenses (Cassells and Curry, 2001; Fehér, 2015; Zhou et al., 2016). Dihydroflavonols and proanthocyanidins possess strong antioxidant properties (Brunetti et al., 2013), and their accumulation may provide redox buffering capacity, protecting cells from oxidative damage associated with dedifferentiation (Zhou et al., 2016; Huang et al., 2019).

The strong upregulation of *F3’5’H* and the preferential accumulation of its products—highly hydroxylated flavonoids such as dihydromyricetin—further support this interpretation. High levels of B-ring hydroxylation are typically associated with enhanced antioxidant capacity (Agati et al., 2012), indicating that both genotypes synthesize more potent antioxidant metabolites as part of their dedifferentiation-associated stress response.

Similar patterns of flavonoid accumulation during SE have been reported across diverse plant species, including Korean pine (Peng et al., 2022), *Gossypium hirsutum* (Guo et al., 2019), *Paeonia ostii* (Zhang et al., 2023), and *Silybum marianum* (Khan et al., 2015), where flavonoids were suggested to promote differentiation and create a more favorable cellular environment for embryo formation. Collectively, these findings support a conserved role of flavonoid-mediated antioxidant activity during the initial phases of somatic embryogenesis.

### 4.3 Metabolic divergence during the callus-to-differentiation transition distinguishes embryogenic competence

In contrast to the conserved metabolic responses observed during early dedifferentiation, the callus-to-differentiation transition was characterized by striking metabolic divergence between the two genotypes. This divergence corresponded closely with their contrasting embryogenic capacities.

The HE genotype adopted a conversion-type metabolic pattern during the HE-IC vs. HE-EC transition. From the downregulation of the upstream rate-limiting enzyme *CHS* to the broad suppression of downstream glycosylation machinery, the flavonoid biosynthesis pathway was systematically repressed at the transcriptional level, accompanied by an overall attenuation of metabolite accumulation, despite the persistence of selected intermediates. This reconfiguration is consistent with KEGG enrichment results, which showed that the HE genotype preferentially activated primary metabolic pathways essential for growth and development, including valine, leucine and isoleucine biosynthesis and pantothenate and CoA biosynthesis.

By contrast, the LE genotype exhibited a maintenance-type metabolic pattern. Although the biosynthesis rate of anthocyanin precursors declined during differentiation, the glycosylation system became further activated, maintaining a relatively elevated level of secondary metabolic activity compared with the HE genotype, despite partial attenuation during differentiation. This pattern aligned with the enrichment of amino sugar and nucleotide sugar metabolism and biosynthesis of nucleotide sugar*s* in the LE genotype, suggesting a metabolic configuration that favors secondary metabolism even as the tissue fails to undergo somatic embryo formation.

These contrasting patterns likely reflect distinct metabolic resource allocation strategies. The condensation reaction catalyzed by *CHS* requires malonyl-CoA (Winkel-Shirley, 2001; Austin and Noel, 2003), which is also a critical precursor for fatty acid biosynthesis (Rawsthorne, 2002). Developing somatic embryos demand substantial lipid production to support rapid cell division and membrane biogenesis (Baud and Lepiniec, 2010; Troncoso-Ponce et al., 2011). Thus, persistent high CHS expression in the LE genotype may create competition with lipid metabolism for shared precursors, potentially constraining embryo development. In contrast, the downregulation of *CHS* in the HE genotype during differentiation may alleviate this competition, thereby redirecting carbon resources toward primary metabolic processes necessary for embryogenesis.

Similarly, the widespread suppression of the glycosylation system in the HE genotype likely increases the availability of nucleotide sugar precursors. Glycosylation reactions consume substantial amounts of UDP-glucose, a central metabolic hub involved in a range of biosynthetic pathways (Bar-Peled and O’Neill, 2011). UDP-glucose serves not only as a glycosyl donor for secondary metabolites (Vogt and Jones, 2000), but also as the direct substrate for cellulose synthesis (Delmer, 1999). Through UDP-glucose dehydrogenase, it can be converted to UDP-glucuronic acid and subsequently to UDP-xylose and UDP-arabinose for hemicellulose and pectin biosynthesis (Lim and Bowles, 2004; Lerouxel et al., 2006). Allocation of UDP-glucose to flavonoid glycosylation may therefore compete with cell wall biosynthesis, potentially restricting cell division and tissue organization required for embryo development. The dual repression of precursor synthesis and glycosylation in the HE genotype could thus increase the pool of UDP-glucose and other nucleotide sugars available for primary cell wall formation during embryogenesis.

The concept of a metabolic trade-off between secondary metabolism and embryogenic capacity has been reported in several conifer and angiosperm SE systems. In *Pseudotsuga menziesii*, 48 transcripts associated with flavonoid metabolism were strongly upregulated (up to 34-fold) in non-embryogenic callus (NEC), accompanied by tissue browning and a complete loss of embryogenic competence (Gautier et al., 2018; Gautier et al., 2019). The authors suggested that the characteristic features of NEC included the upregulation of stress response metabolites and a shift in carbohydrate metabolism toward starch storage, indicating that excessive secondary metabolic activity may interfere with the coordinated metabolic patterns required for efficient cell differentiation. A similar conclusion was drawn in *Larix kaempferi*, where 39% of DAMs were flavonoids and phenolic acids predominantly accumulated in NEC, suggesting that excessive secondary metabolism may interfere with primary metabolism and delay differentiation (Wang et al., 2022). In *Paeonia ostii*, flavonoid-related genes—including F3’5’H and ANS—were downregulated in EC and SE relative to NEC (Zhang et al., 2023). Moreover, addition of activated charcoal, which adsorbs phenolics, improved embryogenic callus induction and somatic embryo development in *Gossypium hirsutum* (Kouakou et al., 2007; Thomas, 2008), suggesting that the excessive accumulation of phenolic compounds (including flavonoids) may have an inhibitory effect.

It should be noted that the metabolic differentiation and morphological differentiation in our time-course analysis showed relatively synchronized patterns, with no significant metabolic changes preceding morphological changes. This suggests that changes in flavonoid metabolism may be coordinated with other developmental processes.

### 4.4 The role of flavonoid biosynthesis in SE: a complex regulatory network

A central question emerging from our data is whether the differential regulation of the flavonoid biosynthesis pathway is a cause, a consequence, or a modulator of embryogenic capacity. While our analyses revealed strong associations between metabolic divergence and developmental outcomes, the underlying causal relationships remain unresolved.

Several studies suggest that upstream developmental regulators may coordinate metabolic reprogramming during SE. Key transcription factors such as BBM, WUS, and LEC1 are well-established determinants of embryogenic competence (Braybrook and Harada, 2008; Horstman et al., 2017). In *Larix kaempferi*, transcriptome profiling and predicted gene regulatory networks identified specific ERF, MYB, and DOF transcription factors as potential regulators of phenylpropanoid and flavonoid biosynthesis (Wang et al., 2022). MYB proteins are known to form MBW complexes with bHLH and WD40 partners to modulate flavonoid pathway gene expression (Li, 2014; Khan et al., 2015). These findings suggest that differences in upstream transcriptional regulatory networks associated with embryogenic competence may indirectly influence SE outcomes by coordinating the expression of multiple developmental and metabolic pathways, including flavonoid biosynthesis.

Conversely, metabolic states may exert feedback effects on developmental capacity. Several mechanisms have been proposed: (1) Resource competition, as discussed above, where flavonoid biosynthesis competes with primary metabolic pathways for shared precursors. (2) Modulation of hormone signaling and polar auxin transport, mediated by flavonoid effects on auxin efflux carriers and related regulatory proteins (Peer and Murphy, 2007). (3) Changes in cellular redox homeostasis, which influence signal transduction. In *Picea* species, a reduced redox state promotes somatic embryo induction, whereas a more oxidized environment favors embryo differentiation and development (Stasolla et al., 2003b). Furthermore, disturbances in ROS homeostasis interact with auxin signaling to regulate SE (Zhou et al., 2016; Kudełko and Gaj, 2019).

Although these mechanisms suggest plausible links between flavonoid accumulation and SE competence, direct experimental evidence connecting specific flavonoid species to embryogenesis remains limited.

Based on available findings, we propose that the relationship between flavonoid metabolism and embryogenic capacity represents a dynamic, bidirectional regulatory system rather than a linear causal pathway. Initial embryogenic competence—shaped by genetic and epigenetic factors—may trigger specific metabolic reprogramming events, including activation or suppression of flavonoid biosynthesis. In turn, these metabolic changes influence cell physiology through multiple layers, such as resource allocation, hormone transport, and redox balance, thereby positively or negatively affecting the progression of embryogenic development. Such reciprocal interactions may amplify small initial differences in competence into pronounced developmental outcomes.

In the context of our experimental system, one possible interpretation is that timely suppression of flavonoid biosynthesis in the HE genotype during the callus-to-differentiation transition reduces carbon and energy diversion into secondary metabolism, thereby favoring the biosynthetic demands of primary metabolism required for embryo formation. This could contribute to a self-reinforcing positive feedback loop that supports successful somatic embryogenesis. In contrast, the LE genotype maintains high levels of secondary metabolism, potentially causing persistent carbon and precursor diversion that interferes with the metabolic restructuring necessary for embryogenic development.

However, we emphasize that these interpretations remain hypothetical. Definitive resolution of the causal connections between flavonoid metabolism and embryogenic capacity will require targeted experiments, such as metabolic flux analysis, exogenous application or chemical inhibition of specific flavonoids, and isotope tracing to directly test how metabolic pathways influence SE outcomes. Future studies combining targeted manipulation of key DAMs (e.g., polyamine conjugates, flavonoid aglycones) with transcription factor binding assays (such as ChIP-qPCR for BBM, LEC1, or MYB regulators) will be essential to establish direct links between transcriptional regulation, metabolic flux, and embryogenic competence.

### 4.5 Functional implications of key differentially accumulated metabolites

In addition to pathway-level differences, several individual DAMs showed pronounced stage- or genotype-specific accumulation patterns, suggesting potential functional roles during SE. Although their precise mechanisms remain unclear, these metabolites represent important clues to the metabolic framework underlying embryogenic competence.

N1,N10-bis(p-coumaroyl)spermidine, which accumulated across multiple stages in the HE genotype, is a derivative of polyamines such as spermidine and spermine—molecules known to be essential for plant growth, development, and in particular for cell division and differentiation (Hasegawa et al., 1984; Kusano et al., 2008). Acylation of polyamines can alter their biological activity and cellular localization (Bassard et al., 2010). In *Arabidopsis*, polyamine acyltransferases participate in floral organ development (Grienenberger et al., 2009). The specific accumulation of this compound in the HE genotype may therefore reflect enhanced polyamine-related regulatory activity supporting its strong proliferative and differentiation capacity.

Vomifoliol, a degradation product of ABA and carotenoid oxidative cleavage (Hasegawa et al., 1984), accumulated specifically in LE-IC vs. LE-C. ABA plays a critical role in SE, particularly in embryo maturation. Exogenous ABA promotes SE maturation in *Quercus suber* and *Vitis vinifera*, increasing dry mass accumulation (Garcia-Martin et al., 2005; Acanda et al., 2020). In *Arabidopsis*, ABA has been reported to play complex and sometimes contrasting roles in SE. Some studies indicate that modulation of ABA signaling affects SE efficiency, with ABA receptor overexpression repressing SE and ABA-deficient mutants showing increased SE capacity (Chen et al., 2021). Vomifoliol accumulation may therefore indicate altered ABA metabolism or signaling in the LE genotype during differentiation.

The LE genotype also exhibited significant accumulation of purine metabolism intermediates, including xanthine and hypoxanthine. These compounds arise from nucleotide catabolism but can also be recycled through salvage pathways for nucleotide synthesis (Ashihara et al., 2018). Purine nucleotides are essential for DNA/RNA synthesis, ATP-driven energy metabolism, and signaling (Stasolla et al., 2003a). Studies in *Picea glauca* SE showed that proliferating cells rely on both *de novo* and salvage pathways to meet nucleotide demand (Stasolla and Thorpe, 2004). The accumulation of these catabolic intermediates in the LE genotype may reflect an imbalance in nucleotide metabolism.

L-ascorbic acid (vitamin C) was upregulated during the HE-EC vs. HE-CE stage. As one of the most abundant and functionally important soluble antioxidants in plants, ascorbic acid participates in reactive oxygen species (ROS) scavenging, regeneration of glutathione and other antioxidants, and serves as a cofactor for various dioxygenases, thereby playing essential roles in plant development and stress responses (Ishikawa et al., 2018; Celi et al., 2023; Conklin et al., 2024). In plant tissue culture systems, numerous studies have demonstrated that ascorbic acid profoundly influences somatic embryogenesis and regeneration. It effectively suppresses the oxidation of phenolic compounds released from wounded explants, thereby reducing tissue browning and cell death, which enhances explant survival and the viability of embryogenic cells (Amente and Chimdessa, 2021; Wang et al., 2024). Moreover, modulation of the glutathione/ascorbate redox state has been shown to significantly affect the efficiency of embryogenic callus induction, the establishment of embryo polarity, and the formation of meristematic primordia (Stasolla, 2010; Ishikawa et al., 2018; Conklin et al., 2024). Therefore, we hypothesize that the upregulation of L-ascorbic acid during somatic embryo formation in the HE genotype may help protect rapidly dividing embryogenic cells from ROS-induced damage and facilitate cell-wall remodeling and other growth processes associated with embryo patterning, ultimately supporting normal somatic embryo development and meristem formation.

### 4.6 Significance and future perspectives

Our findings have practical implications for improving somatic embryogenesis efficiency in *H. brasiliensis* and potentially other recalcitrant species within the experimental framework examined here. Identifying the callus-to-differentiation transition as a critical metabolic checkpoint suggests that interventions during this stage are likely to be most effective. Definitive resolution of causal relationships within this framework will require targeted functional validation. In particular, genetic manipulation of key flavonoid biosynthetic nodes such as CHS and flavonoid glycosyltransferases, together with *in vitro* supplementation or inhibition assays, would provide a direct means to test whether modulation of flavonoid flux influences embryogenic competence.

The integration of metabolomic and transcriptomic datasets highlights the importance of evaluating metabolic phenotypes, not only gene expression patterns, when optimizing SE systems. Medium composition may be refined to favor primary metabolism over secondary metabolism during the callus-to-differentiation transition—for example, through adjusting nitrogen sources, carbon-nitrogen ratios, or supplementing metabolic intermediates that redirect flux away from the phenylpropanoid pathway. It should be noted that the metabolic reprogramming captured in this study likely represents downstream outputs of broader transcriptional regulation during SE. Key embryogenesis-associated transcription factors, such as BBM and LEC, are known to orchestrate large-scale developmental transitions and may indirectly shape the observed accumulation patterns of core DAMs through regulation of hormone- and redox-related genes. Future integration of transcription factor binding information with transcriptomic and metabolomic datasets would facilitate the construction of a conceptual “transcription factor–gene–metabolite” regulatory network, providing a systems-level framework for linking developmental regulators with metabolic state transitions during somatic embryogenesis.

Several questions remain. First, the causal relationship between flavonoid metabolism and embryogenic capacity requires direct experimental validation, such as genetic manipulation or chemical inhibition of specific flavonoids. Second, the proposed resource competition model involving malonyl-CoA, UDP-glucose, and other precursors remains theoretical without supporting metabolic flux evidence. Third, the upstream regulators that coordinate multiple metabolic programs are still unknown, and may involve higher-order regulatory layers such as epigenetic mechanisms, including chromatin accessibility, DNA methylation, and histone modification. Finally, while flavonoid biosynthesis emerged as the pathway with the most consistent enrichment, other metabolic processes—including amino acid, nucleotide, and lipid metabolism—also differed between genotypes and may play important roles.

Importantly, these metabolic classes should not be viewed as independent of flavonoid metabolism but as interconnected components of a coordinated metabolic reprogramming underlying somatic embryogenesis. Amino acid, nucleotide, lipid, polyamine, and antioxidant pathways are metabolically linked to phenylpropanoid metabolism through shared carbon precursors, redox homeostasis, and cellular energy demands, and their genotype- and stage-specific alterations likely reflect shifts in resource allocation accompanying the acquisition of embryogenic competence.

In this context, flavonoid biosynthesis emerged as the most consistently enriched and clearly differentiated pathway between genotypes, justifying its detailed discussion in this study. Nevertheless, the accumulation patterns of non-flavonoid metabolites indicate that embryogenic potential is regulated by an integrated metabolic network rather than by a single compound class, with flavonoids representing a prominent but not exclusive node within this framework. Future studies should therefore focus on how multiple metabolic pathways are integrated and co-regulated during somatic embryogenesis, rather than on individual pathways in isolation.

Although the present study focused on two genotypes with contrasting embryogenic capacities using a single explant type, this design enabled clear resolution of genotype-dependent metabolic divergence under highly controlled developmental conditions. Embryogenic competence, however, is likely a quantitative trait rather than a binary state, and inclusion of genotypes with intermediate embryogenic capacity will be important for determining whether the observed suppression of flavonoid biosynthesis during the callus-to-differentiation transition follows a gradual or threshold-dependent pattern. Furthermore, because somatic embryogenesis can be initiated from diverse explant sources with distinct physiological and stress-response backgrounds, comparative metabolomic analyses across different explant types (e.g., shoot tips, leaves) will be valuable for assessing whether flavonoid-associated metabolic reprogramming reflects a general regulatory principle or an explant-specific response. Such expanded experimental frameworks will help define the broader applicability of the metabolic trade-offs proposed in this study.

## Data Availability

The datasets presented in this study can be found in online repositories. The names of the repository/repositories and accession number(s) can be found in the article/**Supplementary Material**.

## Glossary

ABA: abscisic acid
*CHS*: *chalcone synthase*
*CHI*: *chalcone isomerase*
CV: coefficient of variation
DAMs: differentially accumulated metabolites
DEGs: differentially expressed genes
*F3H*: *flavanone 3-hydroxylase*
*F3’5’H*: *flavonoid 3′,5′-hydroxylase*
*FLS*: *flavonol synthase*
HE: high-embryogenic
HE-CE: cotyledonary embryo stage of high-embryogenic genotype
HE-EC: embryogenic callus stage of high-embryogenic genotype
HE-EX: explant stage of high-embryogenic genotype
HE-IC: induced callus stage of high-embryogenic genotype
KEGG: Kyoto Encyclopedia of Genes and Genomes
LE: low-embryogenic
LE-C: non-embryogenic callus of low-embryogenic genotype
LE-EX: explant stage of low-embryogenic genotype
LE-IC: induced callus stage of low-embryogenic genotype
*LAR*: *leucoanthocyanidin reductase*
OPLS-DA: orthogonal projections to latent structures discriminant analysis
PCA: principal component analysis
ROS: reactive oxygen species
SE: somatic embryogenesis.
