# Supplementary material for: Stage-specific metabolic divergence in flavonoid biosynthesis correlates with embryogenic capacity in rubber tree (Hevea brasiliensis)
Source: Front Plant Sci. 2026 Feb 4;17:1766162. doi: 10.3389/fpls.2026.1766162 (PMC12913399; doi:10.3389/fpls.2026.1766162)
Supplement: Supplementary file 1 [file DataSheet1.docx]

**Stage-specific metabolic divergence in flavonoid biosynthesis correlates with embryogenic capacity in rubber tree (*Hevea brasiliensis*)**

**Jia Miao^1^, Xiao-Long Sun^1^, Jin Liu ^1^, Ming-Chun Gui^1^, Min Tang^1^, Hai Tian^1^, Wan-Yuan Shi^1^and Ling Li^*^**

^1^ Yunnan Key Laboratory of Sustainable Utilization Research on Rubber Tree, the Center of Rubber Research, Yunnan Institute of Tropical Crops, Xishuangbanna, 666100, China

**Corresponding authors:** Ling Li, liling2612@163.com

**Supplementary Information**

Supplementary Table S1: Top 10 DAMs across eight pairwise comparisons.

Supplementary Table S2: DEGs enriched in the flavonoid biosynthesis pathway in the LE-EX vs. LE-IC comparison.

Supplementary Table S3: DEGs enriched in the flavonoid biosynthesis pathway in the HE-EX vs. HE-IC comparison.

Supplementary Table S4: DAMs enriched in the flavonoid biosynthesis pathway in the LE-EX vs. LE-IC comparison.

Supplementary Table S5: DAMs enriched in the flavonoid biosynthesis pathway in the HE-EX vs. HE-IC comparison.

Supplementary Table S6: DEGs enriched in the flavonoid biosynthesis pathway in the LE-IC vs. LE-C comparison.

Supplementary Table S7: DEGs enriched in the flavonoid biosynthesis pathway in the HE-IC vs. HE-EC comparison.

Supplementary Table S8: DAMs enriched in the flavonoid biosynthesis pathway in the LE-IC vs. LE-C comparison.

Supplementary Table S9: DAMs enriched in the flavonoid biosynthesis pathway in the HE-IC vs. HE-EC comparison.

Supplementary Figure S1: Distribution of coefficient of variation (CV) values across all samples.

Supplementary Figure S2: Integrated KEGG pathway visualization of flavonoid biosynthesis in the HE-EC vs. HE-CE comparison.

Supplementary Figure S3: KEGG enrichment analysis of DEGs and DAMs.

Supplementary Figure S4: Replicate-level heatmaps supporting the flavonoid pathway analysis in Figure 6.

Supplementary Figure S5: Replicate-level heatmaps supporting the flavonoid pathway analysis during differentiation in Figure 8.

**Supplementary Table S1**. Top 10 differential accumulated metabolites (DAMs) across all pairwise comparisons. This table summarizes the top ten significantly differential accumulated metabolites identified in each comparison group (LE-EX vs. HE-EX, LE-IC vs. HE-IC, LE-C vs. HE-EC, LE-EX vs. LE-IC, LE-IC vs. LE-C, HE-EX vs. HE-IC, HE-IC vs. HE-EC, and HE-EC vs. HE-CE). For each metabolite, the metabolite ID, molecular formula, compound name, metabolite class (Class I and Class II), VIP score, *p*-value, log₂ fold change (Log₂FC), and regulation direction (up/down) are provided.

| Group | Metabolite ID | Formula | Compounds | Class I | Class II | VIP | *p*-value | Log_2_FC | Direction |
| --- | --- | --- | --- | --- | --- | --- | --- | --- | --- |
| LE-EX vs HE-EX | pme2828 | C6H5NO3 | 4-Nitrophenol | Phenolic acids | Phenolic acids | 1.27 | 0.0006 | 18.01 | up |
| LE-EX vs HE-EX | pmb0770 | C20H20N2O4 | N-Feruloylserotonin | Alkaloids | Plumerane | 1.27 | 0.0231 | 12.51 | up |
| LE-EX vs HE-EX | pmb3072 | C22H26O12 | 3-O-p-Coumaroylshikimic acid-O-glucoside | Phenolic acids | Phenolic acids | 1.27 | 0.0418 | 12.12 | up |
| LE-EX vs HE-EX | mws0056 | C21H20O12 | Myricetin-3-O-rhamnoside (Myricitrin) | Flavonoids | Flavonols | 1.27 | 0.0019 | 7.60 | up |
| LE-EX vs HE-EX | mws1045 | C27H30O17 | Myricetin-3-O-rutinoside | Flavonoids | Flavonols | 1.27 | 0.0048 | 4.32 | up |
| LE-EX vs HE-EX | Lmhn003223 | C13H12O7 | Cinnamoyltartaric acid | Phenolic acids | Phenolic acids | 1.27 | 0.0434 | -10.25 | down |
| LE-EX vs HE-EX | Lmhn102068 | C13H12O8 | 2-O-Caffeoylmalic acid | Phenolic acids | Phenolic acids | 1.27 | 0.0411 | -10.34 | down |
| LE-EX vs HE-EX | Lmbn001034 | C6H12O4 | 2,3-Dihydroxy-3-methylpentanoic acid | Organic acids | Organic acids | 1.27 | 0.0319 | -12.88 | down |
| LE-EX vs HE-EX | Lmdp003146 | C10H14O2 | Rhododendrol | Phenolic acids | Phenolic acids | 1.27 | 0.0212 | -16.59 | down |
| LE-EX vs HE-EX | Lssp210344 | C26H42O10 | Craiobioside B | Others | Saccharides | 1.27 | 0.0303 | -17.45 | down |
| LE-IC vs HE-IC | pma0692 | C25H31N3O4 | N1,N10-Bis(p-coumaroyl)spermidine | Alkaloids | Phenolamine | 1.29 | 0.0011 | 15.88 | up |
| LE-IC vs HE-IC | Lssp210056 | C21H20O12 | Quercetin3-O-galactoside | Flavonoids | Other Flavonoids | 1.29 | 0.0397 | 15.51 | up |
| LE-IC vs HE-IC | pme1975 | C3H4O4 | Malonic acid | Organic acids | Organic acids | 1.29 | 0.0293 | 13.36 | up |
| LE-IC vs HE-IC | pma0170 | C27H35N3O6 | N',N''-Diferuloylspermidine | Alkaloids | Phenolamine | 1.29 | 0.0040 | 12.19 | up |
| LE-IC vs HE-IC | mws4085 | C11H12O5 | Sinapic acid | Phenolic acids | Phenolic acids | 1.29 | 0.0283 | 11.59 | up |
| LE-IC vs HE-IC | pmn001525 | C23H26O11 | 3,5-Digalloylshikimic acid | Phenolic acids | Phenolic acids | 1.29 | 0.0035 | 11.27 | up |
| LE-IC vs HE-IC | Lmcp004624 | C16H12O6 | 3-Methylkaempferol* | Flavonoids | Flavonols | 1.29 | 0.0435 | -12.49 | down |
| LE-IC vs HE-IC | Lmjp003044 | C22H22O12 | Isorhamnetin-3-O-Glucoside | Flavonoids | Flavonols | 1.29 | 0.0251 | -12.51 | down |
| LE-IC vs HE-IC | Lmqp000427 | C6H11NO3 | N-Methyl-Trans-4-Hydroxy-L-Proline | Amino acids and derivatives | Amino acids and derivatives | 1.29 | 0.0108 | -12.99 | down |
| LE-IC vs HE-IC | Qmjp081208 | C21H30O8 | Sarcaglaboside A | Terpenoids | Sesquiterpenoids | 1.29 | 0.0371 | -13.55 | down |
| LE-C vs HE-EC | MWSslk177 | C14H19NO8 | Lithospermoside | Alkaloids | Alkaloids | 1.22 | 0.0073 | 17.14 | up |
| LE-C vs HE-EC | pme0368 | C27H30O14 | Apigenin-7-O-rutinoside (Isorhoifolin) | Flavonoids | Flavones | 1.22 | 0.0110 | 15.15 | up |
| LE-C vs HE-EC | mws1002 | C17H14O8 | Syringetin | Flavonoids | Flavonols | 1.22 | 0.0086 | 12.56 | up |
| LE-C vs HE-EC | pmp000695 | C16H22O10 | Swertiamarin | Terpenoids | Monoterpenoids | 1.22 | 0.0287 | 12.25 | up |
| LE-C vs HE-EC | Lmlp003531 | C21H20O11 | Luteolin-3'-O-glucoside* | Flavonoids | Flavones | 1.22 | 0.0227 | 11.99 | up |
| LE-C vs HE-EC | pmp000194 | C24H22O13 | 6''-O-Malonylgenistin | Flavonoids | Isoflavones | 1.22 | 0.0210 | -12.44 | down |
| LE-C vs HE-EC | Hmcp001578 | C28H32O17 | Isorhamnetin-3,7-O-diglucoside | Flavonoids | Flavonols | 1.22 | 0.0290 | -13.85 | down |
| LE-C vs HE-EC | Qmyp101232 | C13H20O3 | Vomifoliol | Terpenoids | Monoterpenoids | 1.22 | 0.0052 | -14.87 | down |
| LE-C vs HE-EC | pmp000413 | C21H20O10 | Genistein-8-C-glucoside | Flavonoids | Isoflavones | 1.22 | 0.0067 | -15.43 | down |
| LE-C vs HE-EC | Hmcp002187 | C23H24O13 | Limocitrin-3-O-galactoside | Flavonoids | Flavonols | 1.22 | 0.0018 | -17.68 | down |
| LE-EX vs LE-IC | pme2828 | C6H5NO3 | 4-Nitrophenol | Phenolic acids | Phenolic acids | 1.11 | 0.0001 | 21.16 | up |
| LE-EX vs LE-IC | Wafp002310 | C15H20N2O5 | 1-O-Caffeoyllysine | Alkaloids | Phenolamine | 1.11 | 0.0108 | 19.25 | up |
| LE-EX vs LE-IC | NK10264324 | C6H6O3 | Phloroglucinol; 1,3,5-Benzenetriol | Phenolic acids | Phenolic acids | 1.11 | 0.0010 | 19.14 | up |
| LE-EX vs LE-IC | Lmmn003663 | C14H18O9 | 5-Glucosyloxy-2-Hydroxybenzoic acid methyl ester | Phenolic acids | Phenolic acids | 1.11 | 0.0007 | 18.32 | up |
| LE-EX vs LE-IC | pmf0116 | C27H31O17+ | Delphinidin-3,5-di-O-glucoside | Flavonoids | Anthocyanidins | 1.11 | 0.0023 | 18.00 | up |
| LE-EX vs LE-IC | Cmmn013378 | C20H30O2 | Levopimaric acid* | Terpenoids | Ditepenoids | 1.11 | 0.0046 | 17.96 | up |
| LE-EX vs LE-IC | Lmcp000282 | C7H15NO3 | L-Carnitine | Alkaloids | Alkaloids | 1.11 | 0.0004 | -17.96 | down |
| LE-EX vs LE-IC | Lmzn006795 | C30H46O3 | 3-Oxours-12-en-28-oic acid (Ursonic acid)* | Terpenoids | Triterpene | 1.11 | 0.0061 | -18.28 | down |
| LE-EX vs LE-IC | Lmcp004436 | C30H27O14+ | Cyanidin-3-O-(6''-O-caffeoyl)glucoside | Flavonoids | Anthocyanidins | 1.11 | 0.0120 | -21.61 | down |
| LE-EX vs LE-IC | pmb3012 | C22H22O11 | Chrysoeriol-7-O-glucoside | Flavonoids | Flavones | 1.11 | 0.0006 | -23.05 | down |
| LE-IC vs LE-C | pma0692 | C25H31N3O4 | N1,N10-Bis(p-coumaroyl)spermidine | Alkaloids | Phenolamine | 1.22 | 0.0012 | 17.01 | up |
| LE-IC vs LE-C | Lmcp000282 | C7H15NO3 | L-Carnitine | Alkaloids | Alkaloids | 1.22 | 0.0009 | 15.92 | up |
| LE-IC vs LE-C | pme1975 | C3H4O4 | Malonic acid | Organic acids | Organic acids | 1.22 | 0.0089 | 14.32 | up |
| LE-IC vs LE-C | mws1002 | C17H14O8 | Syringetin | Flavonoids | Flavonols | 1.22 | 0.0002 | -13.08 | down |
| LE-IC vs LE-C | Lmyp003348 | C28H24O15 | Kaempferol-3-O-(6''-galloyl)galactoside* | Flavonoids | Flavonols | 1.22 | 0.0105 | -14.15 | down |
| LE-IC vs LE-C | pme2459 | C21H20O11 | Luteolin-7-O-glucoside (Cynaroside) | Flavonoids | Flavones | 1.22 | 0.0341 | -14.40 | down |
| LE-IC vs LE-C | HJN089 | C21H20O10 | Sophoricoside | Flavonoids | Isoflavones | 1.22 | 0.0050 | -14.41 | down |
| LE-IC vs LE-C | pmp000695 | C16H22O10 | Swertiamarin | Terpenoids | Monoterpenoids | 1.22 | 0.0221 | -15.18 | down |
| LE-IC vs LE-C | HJAP026 | C28H32O16 | Chrysoeriol-6-C-glucoside-4'-O-glucoside | Flavonoids | Flavones | 1.22 | 0.0172 | -16.04 | down |
| LE-IC vs LE-C | pme0368 | C27H30O14 | Apigenin-7-O-rutinoside (Isorhoifolin) | Flavonoids | Flavones | 1.22 | 0.0369 | -16.88 | down |
| HE-EX vs HE-IC | Hmln000873 | C13H16O10 | 2-O-Galloyl-D-glucose | Phenolic acids | Phenolic acids | 1.09 | 0.0005 | 20.75 | up |
| HE-EX vs HE-IC | Lssp210344 | C26H42O10 | Craiobioside B | Others | Saccharides | 1.09 | 0.0048 | 20.61 | up |
| HE-EX vs HE-IC | NK10264324 | C6H6O3 | Phloroglucinol; 1,3,5-Benzenetriol | Phenolic acids | Phenolic acids | 1.09 | 0.0002 | 19.38 | up |
| HE-EX vs HE-IC | Wafp002310 | C15H20N2O5 | 1-O-Caffeoyllysine | Alkaloids | Phenolamine | 1.09 | 0.0092 | 19.38 | up |
| HE-EX vs HE-IC | Lmmn003663 | C14H18O9 | 5-Glucosyloxy-2-Hydroxybenzoic acid methyl ester | Phenolic acids | Phenolic acids | 1.09 | 0.0005 | 18.02 | up |
| HE-EX vs HE-IC | Lmcp004624 | C16H12O6 | 3-Methylkaempferol* | Flavonoids | Flavonols | 1.09 | 0.0013 | -18.76 | down |
| HE-EX vs HE-IC | Lmjp003044 | C22H22O12 | Isorhamnetin-3-O-Glucoside | Flavonoids | Flavonols | 1.09 | 0.0133 | -19.34 | down |
| HE-EX vs HE-IC | Wafn001957 | C15H16O11 | 2-O-Caffeoylglucaric Acid | Phenolic acids | Phenolic acids | 1.09 | 0.0025 | -19.57 | down |
| HE-EX vs HE-IC | Zbrp003982 | C21H20O10 | Sulfurein; Sulfuretin-6-O-glucoside | Flavonoids | Aurones | 1.09 | 0.0020 | -20.29 | down |
| HE-EX vs HE-IC | pmb3012 | C22H22O11 | Chrysoeriol-7-O-glucoside | Flavonoids | Flavones | 1.09 | 0.0037 | -21.23 | down |
| HE-IC vs HE-EC | Lmqp000427 | C6H11NO3 | N-Methyl-Trans-4-Hydroxy-L-Proline | Amino acids and derivatives | Amino acids and derivatives | 1.24 | 0.0130 | 14.14 | up |
| HE-IC vs HE-EC | Lmgn002184 | C7H10O5 | 2-Oxoheptanedionic acid* | Organic acids | Organic acids | 1.24 | 0.0158 | 13.63 | up |
| HE-IC vs HE-EC | Qmjp081208 | C21H30O8 | Sarcaglaboside A | Terpenoids | Sesquiterpenoids | 1.24 | 0.0102 | 13.62 | up |
| HE-IC vs HE-EC | Lmgp003335 | C21H20O10 | 4',5-Dihydroxyisoflavone-7-O-galactoside | Flavonoids | Isoflavones | 1.24 | 0.0265 | -12.71 | down |
| HE-IC vs HE-EC | mws2209 | C21H20O11 | Kaempferol-3-O-glucoside (Astragalin) | Flavonoids | Flavonols | 1.24 | 0.0074 | -13.60 | down |
| HE-IC vs HE-EC | pmb0665 | C27H30O16 | Orientin-7-O-glucoside | Flavonoids | Flavones | 1.24 | 0.0389 | -14.51 | down |
| HE-IC vs HE-EC | Hmlp001371 | C12H14N2O3 | Cyclo(Tyr-Ala) | Amino acids and derivatives | Amino acids and derivatives | 1.24 | 0.0039 | -14.67 | down |
| HE-IC vs HE-EC | Hmcp001578 | C28H32O17 | Isorhamnetin-3,7-O-diglucoside | Flavonoids | Flavonols | 1.24 | 0.0084 | -14.87 | down |
| HE-IC vs HE-EC | Lssp210056 | C21H20O12 | Quercetin3-O-galactoside | Flavonoids | Other Flavonoids | 1.24 | 0.0397 | -15.51 | down |
| HE-IC vs HE-EC | Hmcp002187 | C23H24O13 | Limocitrin-3-O-galactoside | Flavonoids | Flavonols | 1.24 | 0.0173 | -16.76 | down |
| HE-EC vs HE-CE | Hmcp001578 | C28H32O17 | Isorhamnetin-3,7-O-diglucoside | Flavonoids | Flavonols | 1.13 | 0.0436 | 15.02 | up |
| HE-EC vs HE-CE | Hmlp001371 | C12H14N2O3 | Cyclo(Tyr-Ala) | Amino acids and derivatives | Amino acids and derivatives | 1.13 | 0.0021 | 14.83 | up |
| HE-EC vs HE-CE | Zmhn002508 | C21H28O12 | 4-p-Cumaroyl-rhamnosyl-(1→6)-D-glucose | Phenolic acids | Phenolic acids | 1.13 | 0.0009 | -14.19 | down |
| HE-EC vs HE-CE | Lmjp002906 | C22H22O12 | Rhamnetin-3-O-Glucoside | Flavonoids | Flavonols | 1.13 | 0.0404 | -14.29 | down |
| HE-EC vs HE-CE | Lmjp003295 | C22H22O12 | 6-Methoxykaempferol-3-O-glucoside | Flavonoids | Flavones | 1.13 | 0.0404 | -14.29 | down |
| HE-EC vs HE-CE | Lmyn005006 | C30H28O13 | Eriodictyol-7-O-(6''-O-p-coumaroyl)glucoside | Flavonoids | Flavanones | 1.13 | 0.0326 | -15.04 | down |
| HE-EC vs HE-CE | mws1073 | C27H30O15 | Apigenin-6,8-di-C-glucoside (Vicenin-2) | Flavonoids | Flavones | 1.13 | 0.0121 | -15.22 | down |
| HE-EC vs HE-CE | Lmdp003146 | C10H14O2 | Rhododendrol | Phenolic acids | Phenolic acids | 1.13 | 0.0067 | -15.75 | down |
| HE-EC vs HE-CE | MWSslk177 | C14H19NO8 | Lithospermoside | Alkaloids | Alkaloids | 1.13 | 0.0073 | -17.14 | down |
| HE-EC vs HE-CE | pmb0478 | C21H26O11 | Coumarin O-rutinoside | Lignans and Coumarins | Coumarins | 1.13 | 0.0222 | -19.99 | down |

**Supplementary Table S2.** Differentially expressed genes (DEGs) associated with the flavonoid biosynthesis pathway in the LE-EX vs. LE-IC comparison. This table lists all differentially expressed genes (DEGs) enriched in the flavonoid biosynthesis pathway in the LE-EX vs. LE-IC comparison. For each gene, the Gene ID, NR annotation, log₂ fold change (log₂FC), *p*-value, adjusted *p*-value (Padj), and direction of change (up/down) are provided.

| Gene ID | NR annotation | Log_2_FC | *p*-value | Padj | Direction |
| --- | --- | --- | --- | --- | --- |
| LOC110631846 | flavonoid 3'-monooxygenase-like [Hevea brasiliensis] | -1.49 | 6.45317E-65 | 5.98235E-64 | down |
| LOC110632801 | omega-hydroxypalmitate O-feruloyl transferase-like [Hevea brasiliensis] | -2.64 | 5.26541E-88 | 6.78892E-87 | down |
| LOC110633200 | chalcone synthase-like [Hevea brasiliensis] | -1.61 | 0.00283928 | 0.004074029 | down |
| LOC110633385 | caffeoyl-CoA O-methyltransferase 1-like [Hevea brasiliensis] | 2.17 | 1.2801E-197 | 4.5785E-196 | up |
| LOC110633409 | protein ECERIFERUM 26-like [Hevea brasiliensis] | -3.43 | 6.72591E-59 | 5.72183E-58 | down |
| LOC110633904 | probable chalcone--flavonone isomerase 3 isoform X1 [Hevea brasiliensis] | 4.26 | 8.3526E-211 | 3.3009E-209 | up |
| LOC110634425 | uncharacterized protein LOC110634425 [Hevea brasiliensis] | -1.27 | 3.48519E-55 | 2.77751E-54 | down |
| LOC110635515 | flavonoid 3'-monooxygenase-like [Hevea brasiliensis] | -8.61 | 2.05528E-12 | 5.38342E-12 | down |
| LOC110636257 | flavonoid 3'-monooxygenase [Hevea brasiliensis] | 4.06 | < 1E-300 | < 1E-300 | up |
| LOC110636943 | vinorine synthase-like [Hevea brasiliensis] | -10.27 | 1.44547E-23 | 5.86424E-23 | down |
| LOC110637122 | protein ECERIFERUM 2-like [Hevea brasiliensis] | -1.15 | 2.06096E-08 | 4.25844E-08 | down |
| LOC110637148 | trans-cinnamate 4-monooxygenase [Hevea brasiliensis] | -1.58 | 1.4463E-171 | 4.2361E-170 | down |
| LOC110638079 | bifunctional dihydroflavonol 4-reductase/flavanone 4-reductase-like [Hevea brasiliensis] | 1.04 | 1.83112E-12 | 4.80771E-12 | up |
| LOC110638682 | probable 2-oxoglutarate-dependent dioxygenase At5g05600 [Hevea brasiliensis] | -7.77 | 4.5851E-282 | 2.7846E-280 | down |
| LOC110640000 | leucoanthocyanidin dioxygenase-like [Hevea brasiliensis] | 6.52 | 1.28344E-09 | 2.8673E-09 | up |
| LOC110640236 | probable 2-oxoglutarate-dependent dioxygenase At3g111800 [Hevea brasiliensis] | -9.06 | 3.12212E-35 | 1.72116E-34 | down |
| LOC110640364 | chalcone synthase 2-like [Hevea brasiliensis] | 1.79 | 1.056E-104 | 1.653E-103 | up |
| LOC110640803 | codeine O-demethylase-like [Hevea brasiliensis] | 2.67 | 3.42498E-07 | 6.56029E-07 | up |
| LOC110640804 | protein SRG1-like [Hevea brasiliensis] | 1.75 | 4.7875E-27 | 2.15304E-26 | up |
| LOC110641151 | naringenin,2-oxoglutarate 3-dioxygenase-like [Hevea brasiliensis] | 4.87 | < 1E-300 | < 1E-300 | up |
| LOC110641350 | chalcone synthase 1 [Hevea brasiliensis] | 4.69 | < 1E-300 | < 1E-300 | up |
| LOC110641698 | caffeoyl-CoA O-methyltransferase-like [Hevea brasiliensis] | -1.22 | 1.81908E-68 | 1.78478E-67 | down |
| LOC110642627 | UDP-glycosyltransferase 88F4-like [Hevea brasiliensis] | -9.15 | 1.93925E-14 | 5.59405E-14 | down |
| LOC110642639 | anthocyanidin 3-O-glucosyltransferase 6-like [Hevea brasiliensis] | -2.74 | 2.76927E-08 | 5.68056E-08 | down |
| LOC110642658 | codeine O-demethylase-like [Hevea brasiliensis] | 3.85 | < 1E-300 | < 1E-300 | up |
| LOC110642702 | fatty alcohol:caffeoyl-CoA acyltransferase-like [Hevea brasiliensis] | -3.76 | 1.315E-139 | 2.9577E-138 | down |
| LOC110642838 | naringenin,2-oxoglutarate 3-dioxygenase [Hevea brasiliensis] | 1.89 | 1.0062E-233 | 4.7006E-232 | up |
| LOC110643140 | protein SRG1-like [Hevea brasiliensis] | -1.22 | 1.85906E-68 | 1.82339E-67 | down |
| LOC110643471 | anthocyanidin 3-O-glucosyltransferase 2-like [Hevea brasiliensis] | -1.34 | 0.001192786 | 0.001771115 | down |
| LOC110644250 | protein SRG1-like [Hevea brasiliensis] | 1.02 | 6.00961E-07 | 1.13351E-06 | up |
| LOC110644267 | protein SRG1-like [Hevea brasiliensis] | -2.63 | 4.43979E-50 | 3.24299E-49 | down |
| LOC110645232 | caffeoyl-CoA O-methyltransferase-like [Hevea brasiliensis] | -10.56 | 2.65368E-59 | 2.27116E-58 | down |
| LOC110645776 | putative anthocyanidin reductase [Hevea brasiliensis] | 2.07 | 1.67953E-19 | 5.92385E-19 | up |
| LOC110646442 | probable 2-oxoglutarate-dependent dioxygenase At3g111800 [Hevea brasiliensis] | -1.37 | 5.9332E-64 | 5.41595E-63 | down |
| LOC110647281 | caffeoyl-CoA O-methyltransferase 1-like [Hevea brasiliensis] | 1.21 | 2.63875E-06 | 4.76364E-06 | up |
| LOC110647282 | caffeoyl-CoA O-methyltransferase 1-like [Hevea brasiliensis] | 1.89 | 8.2736E-106 | 1.3131E-104 | up |
| LOC110647691 | vestitone reductase-like isoform X1 [Hevea brasiliensis] | 1.22 | 1.03373E-40 | 6.36759E-40 | up |
| LOC110647757 | non-functional NADPH-dependent codeinone reductase 2-like isoform X1 [Hevea brasiliensis] | 1.16 | 5.96883E-25 | 2.52051E-24 | up |
| LOC110648211 | codeine O-demethylase-like [Hevea brasiliensis] | -3.46 | 3.51806E-59 | 3.00575E-58 | down |
| LOC110648892 | chalcone synthase 2 [Hevea brasiliensis] | 3.47 | < 1E-300 | < 1E-300 | up |
| LOC110649288 | leucoanthocyanidin reductase-like [Hevea brasiliensis] | 1.06 | 7.42796E-41 | 4.58307E-40 | up |
| LOC110649415 | UDP-glycosyltransferase 71K1-like isoform X2 [Hevea brasiliensis] | -2.80 | 2.72402E-53 | 2.10494E-52 | down |
| LOC110649734 | protein ECERIFERUM 2-like isoform X1 [Hevea brasiliensis] | -11.08 | 1.07744E-20 | 3.96861E-20 | down |
| LOC110649802 | S-norcoclaurine synthase 1-like [Hevea brasiliensis] | 4.14 | 1.2058E-146 | 2.9219E-145 | up |
| LOC110650078 | shikimate O-hydroxycinnamoyltransferase-like [Hevea brasiliensis] | -1.31 | 6.10936E-09 | 1.3084E-08 | down |
| LOC110651006 | protein SRG1-like [Manihot esculenta] | -2.63 | 1.5245E-27 | 6.93776E-27 | down |
| LOC110651405 | chalcone--flavonone isomerase 2-like isoform X1 [Hevea brasiliensis] | 2.42 | 2.286E-255 | 1.2148E-253 | up |
| LOC110651918 | leucoanthocyanidin dioxygenase-like [Hevea brasiliensis] | 4.05 | 3.9612E-177 | 1.2115E-175 | up |
| LOC110652765 | licodione synthase-like [Hevea brasiliensis] | -8.80 | 2.61785E-33 | 1.37953E-32 | down |
| LOC110652792 | leucoanthocyanidin dioxygenase-like [Hevea brasiliensis] | 3.77 | < 1E-300 | < 1E-300 | up |
| LOC110653348 | flavonoid 3',5'-hydroxylase 2-like [Hevea brasiliensis] | 6.67 | 1.5055E-159 | 3.9966E-158 | up |
| LOC110653493 | cytochrome P450 CYP73A100-like [Hevea brasiliensis] | 2.93 | 8.6435E-298 | 5.7671E-296 | up |
| LOC110653986 | protein ECERIFERUM 26-like [Hevea brasiliensis] | -8.58 | 5.04983E-68 | 4.9157E-67 | down |
| LOC110654081 | protein ECERIFERUM 26-like [Hevea brasiliensis] | -11.72 | 4.52888E-23 | 1.80609E-22 | down |
| LOC110654731 | UDP-glycosyltransferase 43-like [Hevea brasiliensis] | -2.32 | 3.27486E-99 | 4.82681E-98 | down |
| LOC110655043 | flavonol synthase/flavanone 3-hydroxylase [Hevea brasiliensis] | -4.69 | 2.33839E-29 | 1.11462E-28 | down |
| LOC110655218 | dihydroflavonol 4-reductase-like [Hevea brasiliensis] | 4.27 | < 1E-300 | < 1E-300 | up |
| LOC110655234 | dihydroflavonol 4-reductase-like [Hevea brasiliensis] | 2.53 | 6.3611E-297 | 4.2159E-295 | up |
| LOC110656234 | vestitone reductase-like isoform X1 [Hevea brasiliensis] | -5.46 | 3.66466E-26 | 1.60727E-25 | down |
| LOC110658083 | anthocyanidin reductase ((2S)-flavan-3-ol-forming) [Hevea brasiliensis] | 2.48 | 5.998E-224 | 2.6443E-222 | up |
| LOC110658204 | UDP-glycosyltransferase 88A1-like isoform X1 [Hevea brasiliensis] | 1.98 | 3.7289E-24 | 1.54224E-23 | up |
| LOC110658208 | UDP-glycosyltransferase 88A1-like [Hevea brasiliensis] | 1.87 | 0.000402663 | 0.000620209 | up |
| LOC110658672 | protein SRG1-like [Hevea brasiliensis] | 2.79 | 2.01287E-17 | 6.60407E-17 | up |
| LOC110658726 | anthocyanidin 3-O-glucosyltransferase 1-like [Hevea brasiliensis] | 1.69 | 1.30627E-15 | 3.98646E-15 | up |
| LOC110658731 | anthocyanidin 3-O-glucosyltransferase 2-like isoform X1 [Hevea brasiliensis] | 2.85 | 2.5466E-151 | 6.3629E-150 | up |
| LOC110658732 | anthocyanidin 3-O-glucosyltransferase 2-like [Hevea brasiliensis] | 2.43 | 4.6839E-219 | 1.9883E-217 | up |
| LOC110660132 | vinorine synthase-like [Hevea brasiliensis] | 2.02 | 3.1201E-05 | 5.22233E-05 | up |
| LOC110660191 | BAHD acyltransferase At5g47980-like [Hevea brasiliensis] | 3.89 | 3.28415E-18 | 1.11057E-17 | up |
| LOC110660233 | omega-hydroxypalmitate O-feruloyl transferase-like [Hevea brasiliensis] | -1.87 | 4.6878E-44 | 3.07946E-43 | down |
| LOC110660590 | flavonoid 3',5'-hydroxylase 1-like [Hevea brasiliensis] | 6.03 | 1.62E-169 | 4.6577E-168 | up |
| LOC110660992 | vinorine synthase-like [Hevea brasiliensis] | -2.35 | 0.000141342 | 0.000225345 | down |
| LOC110662985 | protein BRI1-5 ENHANCED 1-like [Hevea brasiliensis] | 2.07 | 2.17023E-08 | 4.47769E-08 | up |
| LOC110662998 | vestitone reductase-like isoform X1 [Hevea brasiliensis] | -4.15 | 2.09223E-15 | 6.32344E-15 | down |
| LOC110663155 | flavonoid 3',5'-hydroxylase 1-like [Hevea brasiliensis] | 3.72 | 2.1911E-117 | 3.9469E-116 | up |
| LOC110663157 | flavonoid 3',5'-hydroxylase 1-like [Hevea brasiliensis] | 3.74 | 2.9696E-73 | 3.137E-72 | up |
| LOC110664234 | protein SRG1-like isoform X2 [Hevea brasiliensis] | 1.06 | 5.00204E-07 | 9.47491E-07 | up |
| LOC110665143 | trans-cinnamate 4-monooxygenase-like [Hevea brasiliensis] | -3.31 | 1.59967E-47 | 1.11692E-46 | down |
| LOC110665349 | licodione synthase-like, partial [Hevea brasiliensis] | -9.41 | 6.85286E-20 | 2.45193E-19 | down |
| LOC110665573 | flavonol synthase/flavanone 3-hydroxylase-like [Hevea brasiliensis] | -9.69 | 1.7674E-188 | 5.8635E-187 | down |
| LOC110666021 | leucoanthocyanidin reductase-like [Hevea brasiliensis] | 2.83 | 3.41973E-71 | 3.49826E-70 | up |
| LOC110666367 | UDP-glycosyltransferase 71K1-like isoform X1 [Hevea brasiliensis] | -1.22 | 4.0593E-05 | 6.7345E-05 | down |
| LOC110666993 | type III polyketide synthase A-like [Hevea brasiliensis] | -12.23 | 1.87949E-48 | 1.33008E-47 | down |
| LOC110667245 | vinorine synthase-like [Hevea brasiliensis] | -2.90 | 1.05991E-06 | 1.96468E-06 | down |
| LOC110667252 | vinorine synthase-like [Hevea brasiliensis] | -6.61 | 3.0035E-136 | 6.5383E-135 | down |
| LOC110667357 | caffeoyl-CoA O-methyltransferase [Hevea brasiliensis] | -1.77 | 6.3791E-122 | 1.2053E-120 | down |
| LOC110668057 | probable chalcone--flavonone isomerase 3 [Hevea brasiliensis] | 2.75 | 5.4026E-217 | 2.2454E-215 | up |
| LOC110668325 | caffeoyl-CoA O-methyltransferase 1-like [Hevea brasiliensis] | 1.17 | 3.62536E-40 | 2.21299E-39 | up |
| LOC110668477 | cytochrome P450 98A2 [Hevea brasiliensis] | -1.28 | 5.215E-110 | 8.6989E-109 | down |
| LOC110668584 | protein SRG1-like [Hevea brasiliensis] | 4.01 | 5.8856E-143 | 1.3641E-141 | up |
| LOC110668970 | type III polyketide synthase B [Hevea brasiliensis] | -8.92 | 1.2261E-102 | 1.8856E-101 | down |
| LOC110670394 | licodione synthase-like [Hevea brasiliensis] | -4.07 | 4.0713E-294 | 2.6627E-292 | down |
| LOC110670575 | UDP-glycosyltransferase 88A1-like [Hevea brasiliensis] | -7.77 | 6.33202E-49 | 4.52626E-48 | down |
| LOC110670639 | UDP-glycosyltransferase 88A1-like [Hevea brasiliensis] | -1.16 | 7.47742E-72 | 7.74767E-71 | down |
| LOC110670671 | UDP-glycosyltransferase 88A1-like [Hevea brasiliensis] | -6.68 | 2.20597E-26 | 9.73676E-26 | down |
| LOC110672953 | phenolic glucoside malonyltransferase 1-like [Hevea brasiliensis] | -5.28 | 1.55168E-41 | 9.69042E-41 | down |
| LOC110673296 | agmatine coumaroyltransferase-2-like [Hevea brasiliensis] | 1.79 | 6.38522E-11 | 1.5423E-10 | up |
| novel.191 | vestitone reductase-like isoform X1 [Hevea brasiliensis] | -3.17 | 2.61671E-12 | 6.82216E-12 | down |
| novel.2231 | protein SRG1-like [Hevea brasiliensis] | -2.17 | 4.99008E-30 | 2.42203E-29 | down |
| novel.2753 | UDP-glycosyltransferase 88B1 [Jatropha curcas] | -2.29 | 4.47776E-19 | 1.56121E-18 | down |
| novel.3247 | anthocyanidin 3-O-glucosyltransferase 2-like [Hevea brasiliensis] | 1.17 | 1.61154E-48 | 1.14263E-47 | up |
| novel.3845 | hypothetical protein MANES_07G031500 [Manihot esculenta] | -3.45 | 4.0103E-105 | 6.3175E-104 | down |

**Supplementary Table S3.** DEGs enriched in the flavonoid biosynthesis pathway in the HE-EX vs. HE-IC comparison.

| Gene ID | NR annotation | log_2_FC | *p*-value | Padj | Direction |
| --- | --- | --- | --- | --- | --- |
| LOC110633200 | chalcone synthase-like [Hevea brasiliensis] | -1.26 | 5.81669E-06 | 1.01301E-05 | down |
| LOC110633409 | protein ECERIFERUM 26-like [Hevea brasiliensis] | -4.59 | 4.96643E-84 | 6.04219E-83 | down |
| LOC110633904 | probable chalcone--flavonone isomerase 3 isoform X1 [Hevea brasiliensis] | 4.46 | 1.4897E-234 | 7.0016E-233 | up |
| LOC110634043 | vinorine synthase-like [Hevea brasiliensis] | 1.90 | 1.36134E-43 | 8.9298E-43 | up |
| LOC110635515 | flavonoid 3'-monooxygenase-like [Hevea brasiliensis] | -5.14 | 7.32673E-12 | 1.83441E-11 | down |
| LOC110636257 | flavonoid 3'-monooxygenase [Hevea brasiliensis] | 3.53 | 4.8591E-264 | 2.8313E-262 | up |
| LOC110636943 | vinorine synthase-like [Hevea brasiliensis] | -9.94 | 6.76954E-17 | 2.1603E-16 | down |
| LOC110637122 | protein ECERIFERUM 2-like [Hevea brasiliensis] | -4.68 | 1.82349E-45 | 1.24362E-44 | down |
| LOC110637148 | trans-cinnamate 4-monooxygenase [Hevea brasiliensis] | -1.66 | 1.16283E-83 | 1.41011E-82 | down |
| LOC110638079 | bifunctional dihydroflavonol 4-reductase/flavanone 4-reductase-like [Hevea brasiliensis] | 1.81 | 5.27144E-41 | 3.27801E-40 | up |
| LOC110638080 | bifunctional dihydroflavonol 4-reductase/flavanone 4-reductase-like isoform X1 [Hevea brasiliensis] | 1.68 | 3.07217E-07 | 5.8281E-07 | up |
| LOC110638621 | GDSL esterase/lipase At4g10955-like [Hevea brasiliensis] | -3.78 | 2.34903E-12 | 6.02826E-12 | down |
| LOC110638682 | probable 2-oxoglutarate-dependent dioxygenase At5g05600 [Hevea brasiliensis] | -10.79 | 8.3922E-155 | 2.1833E-153 | down |
| LOC110640000 | leucoanthocyanidin dioxygenase-like [Hevea brasiliensis] | 8.10 | 1.66861E-11 | 4.10214E-11 | up |
| LOC110640236 | probable 2-oxoglutarate-dependent dioxygenase At3g111800 [Hevea brasiliensis] | -10.33 | 1.06135E-45 | 7.2699E-45 | down |
| LOC110640364 | chalcone synthase 2-like [Hevea brasiliensis] | 3.07 | 2.2354E-298 | 1.6417E-296 | up |
| LOC110640803 | codeine O-demethylase-like [Hevea brasiliensis] | 1.05 | 0.032599881 | 0.041516515 | up |
| LOC110640804 | protein SRG1-like [Hevea brasiliensis] | 1.03 | 6.2517E-11 | 1.48503E-10 | up |
| LOC110641151 | naringenin,2-oxoglutarate 3-dioxygenase-like [Hevea brasiliensis] | 4.12 | < 1E-300 | < 1E-300 | up |
| LOC110641350 | chalcone synthase 1 [Hevea brasiliensis] | 4.30 | < 1E-300 | < 1E-300 | up |
| LOC110641698 | caffeoyl-CoA O-methyltransferase-like [Hevea brasiliensis] | -2.07 | 9.80114E-95 | 1.38261E-93 | down |
| LOC110642610 | shikimate O-hydroxycinnamoyltransferase-like [Hevea brasiliensis] | -1.12 | 6.45098E-40 | 3.92016E-39 | down |
| LOC110642627 | UDP-glycosyltransferase 88F4-like [Hevea brasiliensis] | -9.47 | 4.14969E-15 | 1.22487E-14 | down |
| LOC110642658 | codeine O-demethylase-like [Hevea brasiliensis] | 2.97 | 1.7408E-210 | 7.1284E-209 | up |
| LOC110642702 | fatty alcohol:caffeoyl-CoA acyltransferase-like [Hevea brasiliensis] | -3.91 | 1.72713E-92 | 2.36065E-91 | down |
| LOC110642838 | naringenin,2-oxoglutarate 3-dioxygenase [Hevea brasiliensis] | 3.02 | < 1E-300 | < 1E-300 | up |
| LOC110643140 | protein SRG1-like [Hevea brasiliensis] | -1.64 | 3.50125E-98 | 5.13539E-97 | down |
| LOC110643466 | anthocyanidin 3-O-glucosyltransferase 2-like [Hevea brasiliensis] | -1.73 | 8.0298E-26 | 3.4842E-25 | down |
| LOC110643471 | anthocyanidin 3-O-glucosyltransferase 2-like [Hevea brasiliensis] | -2.70 | 2.50323E-12 | 6.41463E-12 | down |
| LOC110644267 | protein SRG1-like [Hevea brasiliensis] | -7.48 | 5.67261E-82 | 6.71813E-81 | down |
| LOC110645232 | caffeoyl-CoA O-methyltransferase-like [Hevea brasiliensis] | -9.43 | 6.73836E-71 | 6.90515E-70 | down |
| LOC110646442 | probable 2-oxoglutarate-dependent dioxygenase At3g111800 [Hevea brasiliensis] | -4.71 | < 1E-300 | < 1E-300 | down |
| LOC110647693 | vestitone reductase-like [Hevea brasiliensis] | 1.80 | 1.94749E-44 | 1.29972E-43 | up |
| LOC110647757 | non-functional NADPH-dependent codeinone reductase 2-like isoform X1 [Hevea brasiliensis] | 1.10 | 1.3965E-22 | 5.48687E-22 | up |
| LOC110648211 | codeine O-demethylase-like [Hevea brasiliensis] | -3.85 | 2.18628E-100 | 3.29396E-99 | down |
| LOC110648892 | chalcone synthase 2 [Hevea brasiliensis] | 3.34 | < 1E-300 | < 1E-300 | up |
| LOC110649415 | UDP-glycosyltransferase 71K1-like isoform X2 [Hevea brasiliensis] | -2.14 | 2.52109E-62 | 2.29897E-61 | down |
| LOC110649734 | protein ECERIFERUM 2-like isoform X1 [Hevea brasiliensis] | -8.35 | 1.19565E-22 | 4.71013E-22 | down |
| LOC110649753 | anthocyanidin 3-O-glucosyltransferase 2-like [Hevea brasiliensis] | -1.68 | 2.00523E-47 | 1.4167E-46 | down |
| LOC110650078 | shikimate O-hydroxycinnamoyltransferase-like [Hevea brasiliensis] | -1.16 | 3.16153E-09 | 6.77355E-09 | down |
| LOC110651006 | protein SRG1-like [Manihot esculenta] | -5.11 | 4.98029E-70 | 5.05676E-69 | down |
| LOC110651405 | chalcone--flavonone isomerase 2-like isoform X1 [Hevea brasiliensis] | 2.62 | 1.736E-255 | 9.4178E-254 | up |
| LOC110652765 | licodione synthase-like [Hevea brasiliensis] | -8.61 | 3.24978E-24 | 1.34639E-23 | down |
| LOC110652792 | leucoanthocyanidin dioxygenase-like [Hevea brasiliensis] | 2.00 | 4.08822E-69 | 4.10359E-68 | up |
| LOC110653348 | flavonoid 3',5'-hydroxylase 2-like [Hevea brasiliensis] | 5.50 | 5.8605E-294 | 4.1909E-292 | up |
| LOC110653493 | cytochrome P450 CYP73A100-like [Hevea brasiliensis] | 2.13 | 9.784E-143 | 2.2742E-141 | up |
| LOC110653986 | protein ECERIFERUM 26-like [Hevea brasiliensis] | -9.27 | 4.36078E-28 | 2.01972E-27 | down |
| LOC110654081 | protein ECERIFERUM 26-like [Hevea brasiliensis] | -11.12 | 8.49219E-21 | 3.13587E-20 | down |
| LOC110654731 | UDP-glycosyltransferase 43-like [Hevea brasiliensis] | -2.71 | 1.9333E-151 | 4.8603E-150 | down |
| LOC110655043 | flavonol synthase/flavanone 3-hydroxylase [Hevea brasiliensis] | -2.96 | 2.54465E-15 | 7.58373E-15 | down |
| LOC110655218 | dihydroflavonol 4-reductase-like [Hevea brasiliensis] | 2.86 | 2.3979E-221 | 1.0541E-219 | up |
| LOC110655234 | dihydroflavonol 4-reductase-like [Hevea brasiliensis] | 1.79 | 3.747E-102 | 5.7705E-101 | up |
| LOC110656234 | vestitone reductase-like isoform X1 [Hevea brasiliensis] | -8.68 | 2.25889E-12 | 5.80391E-12 | down |
| LOC110658083 | anthocyanidin reductase ((2S)-flavan-3-ol-forming) [Hevea brasiliensis] | 2.70 | 4.5778E-267 | 2.7097E-265 | up |
| LOC110658204 | UDP-glycosyltransferase 88A1-like isoform X1 [Hevea brasiliensis] | 2.07 | 4.1848E-24 | 1.72827E-23 | up |
| LOC110658208 | UDP-glycosyltransferase 88A1-like [Hevea brasiliensis] | 2.07 | 3.39737E-09 | 7.26425E-09 | up |
| LOC110658672 | protein SRG1-like [Hevea brasiliensis] | 1.55 | 0.007252726 | 0.009897666 | up |
| LOC110658723 | anthocyanidin 3-O-glucosyltransferase 1-like [Hevea brasiliensis] | 2.51 | 7.69656E-20 | 2.74541E-19 | up |
| LOC110658731 | anthocyanidin 3-O-glucosyltransferase 2-like isoform X1 [Hevea brasiliensis] | 4.09 | 1.6066E-166 | 4.6535E-165 | up |
| LOC110658732 | anthocyanidin 3-O-glucosyltransferase 2-like [Hevea brasiliensis] | 2.81 | 5.7457E-107 | 9.3648E-106 | up |
| LOC110660136 | acylsugar acyltransferase 3-like [Hevea brasiliensis] | 5.45 | 8.9324E-07 | 1.6404E-06 | up |
| LOC110660191 | BAHD acyltransferase At5g47980-like [Hevea brasiliensis] | 2.57 | 5.76616E-15 | 1.68999E-14 | up |
| LOC110660233 | omega-hydroxypalmitate O-feruloyl transferase-like [Hevea brasiliensis] | -3.33 | 6.4826E-104 | 1.0215E-102 | down |
| LOC110660590 | flavonoid 3',5'-hydroxylase 1-like [Hevea brasiliensis] | 5.20 | 1.896E-177 | 6.0679E-176 | up |
| LOC110660992 | vinorine synthase-like [Hevea brasiliensis] | -5.59 | 3.22448E-12 | 8.21846E-12 | down |
| LOC110662985 | protein BRI1-5 ENHANCED 1-like [Hevea brasiliensis] | 2.14 | 9.26619E-11 | 2.17601E-10 | up |
| LOC110662998 | vestitone reductase-like isoform X1 [Hevea brasiliensis] | -4.19 | 1.86046E-28 | 8.70311E-28 | down |
| LOC110663155 | flavonoid 3',5'-hydroxylase 1-like [Hevea brasiliensis] | 2.69 | 5.7578E-103 | 8.9642E-102 | up |
| LOC110663157 | flavonoid 3',5'-hydroxylase 1-like [Hevea brasiliensis] | 4.44 | 2.5392E-136 | 5.5974E-135 | up |
| LOC110664234 | protein SRG1-like isoform X2 [Hevea brasiliensis] | 2.94 | 4.62177E-19 | 1.60794E-18 | up |
| LOC110665143 | trans-cinnamate 4-monooxygenase-like [Hevea brasiliensis] | -2.54 | 1.11597E-31 | 5.63393E-31 | down |
| LOC110665236 | phenolic glucoside malonyltransferase 1-like [Hevea brasiliensis] | -1.19 | 0.000519149 | 0.000786498 | down |
| LOC110665349 | licodione synthase-like, partial [Hevea brasiliensis] | -10.57 | 8.08891E-19 | 2.78532E-18 | down |
| LOC110665573 | flavonol synthase/flavanone 3-hydroxylase-like [Hevea brasiliensis] | -12.75 | 3.73448E-27 | 1.68525E-26 | down |
| LOC110666021 | leucoanthocyanidin reductase-like [Hevea brasiliensis] | 2.53 | 4.04859E-86 | 5.08276E-85 | up |
| LOC110666367 | UDP-glycosyltransferase 71K1-like isoform X1 [Hevea brasiliensis] | -1.37 | 1.50736E-08 | 3.10061E-08 | down |
| LOC110666993 | type III polyketide synthase A-like [Hevea brasiliensis] | -9.51 | 2.99902E-12 | 7.65033E-12 | down |
| LOC110667245 | vinorine synthase-like [Hevea brasiliensis] | -1.65 | 2.78421E-08 | 5.62905E-08 | down |
| LOC110667252 | vinorine synthase-like [Hevea brasiliensis] | -1.95 | 2.72129E-39 | 1.63573E-38 | down |
| LOC110667357 | caffeoyl-CoA O-methyltransferase [Hevea brasiliensis] | -3.12 | 4.4569E-205 | 1.7693E-203 | down |
| LOC110668057 | probable chalcone--flavonone isomerase 3 [Hevea brasiliensis] | 3.04 | 1.9804E-263 | 1.1495E-261 | up |
| LOC110668477 | cytochrome P450 98A2 [Hevea brasiliensis] | -2.04 | 4.9986E-190 | 1.7558E-188 | down |
| LOC110668970 | type III polyketide synthase B [Hevea brasiliensis] | -6.06 | 7.90218E-50 | 5.82952E-49 | down |
| LOC110669034 | UDP-glycosyltransferase 88F4-like [Hevea brasiliensis] | 3.05 | 2.1872E-08 | 4.45397E-08 | up |
| LOC110670394 | licodione synthase-like [Hevea brasiliensis] | -3.37 | 3.9215E-136 | 8.613E-135 | down |
| LOC110670575 | UDP-glycosyltransferase 88A1-like [Hevea brasiliensis] | -4.99 | 4.07115E-34 | 2.17506E-33 | down |
| LOC110670671 | UDP-glycosyltransferase 88A1-like [Hevea brasiliensis] | -6.48 | 2.8651E-09 | 6.15875E-09 | down |
| LOC110671363 | uncharacterized protein LOC110671363 [Hevea brasiliensis] | 2.41 | 1.93612E-23 | 7.80611E-23 | up |
| LOC110671433 | protein SRG1-like [Hevea brasiliensis] | 5.97 | 2.76326E-06 | 4.91371E-06 | up |
| LOC110672657 | shikimate O-hydroxycinnamoyltransferase-like [Hevea brasiliensis] | -3.61 | < 1E-300 | < 1E-300 | down |
| LOC110672953 | phenolic glucoside malonyltransferase 1-like [Hevea brasiliensis] | -2.90 | 1.13137E-14 | 3.2707E-14 | down |
| LOC110673296 | agmatine coumaroyltransferase-2-like [Hevea brasiliensis] | 3.13 | 7.28662E-24 | 2.9825E-23 | up |
| LOC110673929 | protein SRG1-like [Hevea brasiliensis] | 1.34 | 4.89472E-30 | 2.37753E-29 | up |
| novel.2421 | -- | -5.15 | 9.5551E-10 | 2.122E-09 | down |
| novel.3845 | hypothetical protein MANES_07G031500 [Manihot esculenta] | -3.87 | 1.19274E-88 | 1.5488E-87 | down |

**Supplementary Table S4.** DAMs enriched in the flavonoid biosynthesis pathway in the LE-EX vs. LE-IC comparison.

| Metabolite ID | Compounds | *p*-value | Log_2_FC | Direction | Class I | Class II |
| --- | --- | --- | --- | --- | --- | --- |
| Hmln002806 | 5-O-Caffeoylshikimic acid | 0.0255 | 3.99 | up | Phenolic acids | Phenolic acids |
| Lmyn006227 | Galangin (3,5,7-Trihydroxyflavone) | 0.0372 | -5.91 | down | Flavonoids | Flavones |
| Lmzp002365 | Hesperetin-7-O-glucoside | 0.0008 | -3.28 | down | Flavonoids | Flavanones |
| mws0032 | Myricetin | 0.0028 | 4.31 | up | Flavonoids | Flavonols |
| mws0040 | Chrysin | 0.0607 | -2.91 | down | Flavonoids | Flavones |
| mws0042 | Epigallocatechin | 0.0221 | 4.03 | up | Flavonoids | Flavanols |
| mws0044 | Taxifolin(Dihydroquercetin) | 0.0024 | 4.83 | up | Flavonoids | Flavanonols |
| mws0046 | Naringenin-7-O-Neohesperidoside(Naringin)* | 0.0001 | -3.87 | down | Flavonoids | Flavanones |
| mws0048 | Apigenin-8-C-Glucoside (Vitexin) | 0.0005 | -1.15 | down | Flavonoids | Flavones |
| mws0049 | Gallocatechin | 0.0004 | 3.12 | up | Flavonoids | Flavanols |
| mws0789 | Pinocembrin (Dihydrochrysin) | 0.0011 | 2.95 | up | Flavonoids | Flavanones |
| mws0920 | Tricetin (5,7,3',4',5'-Pentahydroxyflavone) | 0.0003 | -1.28 | down | Flavonoids | Flavones |
| mws1068 | Kaempferol (3,5,7,4'-Tetrahydroxyflavone) | 0.0001 | -3.88 | down | Flavonoids | Flavonols |
| mws1094 | Aromadendrin (Dihydrokaempferol) | 0.0163 | 1.34 | up | Flavonoids | Flavanonols |
| mws1179 | Naringenin-7-O-glucoside (Prunin)* | 0.0016 | -2.73 | down | Flavonoids | Flavanones |
| mws2118 | Phloretin-2'-O-glucoside (Phlorizin) | 0.0047 | 2.60 | up | Flavonoids | Chalcones |
| MWSHY0048 | Dihydromyricetin (Ampelopsin) | 0.0005 | 5.67 | up | Flavonoids | Flavanonols |
| MWSHY0137 | Naringenin (5,7,4'-Trihydroxyflavanone)* | 0.0463 | -3.53 | down | Flavonoids | Flavanones |
| pmb0751 | Trans-5-O-(p-Coumaroyl)shikimate | 0.0008 | 3.42 | up | Phenolic acids | Phenolic acids |
| pmb3074 | 5-O-p-Coumaroylquinic acid* | 0.0901 | 2.84 | up | Phenolic acids | Phenolic acids |
| pme0088 | Luteolin (5,7,3',4'-Tetrahydroxyflavone) | 0.1017 | -7.17 | down | Flavonoids | Flavones |
| pme2960 | Naringenin chalcone; 2',4,4',6'-Tetrahydroxychalcone | 0.0012 | -3.39 | down | Flavonoids | Chalcones |
| pme3475 | Butin; 7,3',4'-Trihydroxyflavanone* | 0.0036 | -3.99 | down | Flavonoids | Flavanones |
| pmp000571 | Apigenin | 0.0100 | -6.20 | down | Flavonoids | Flavones |
| pme1201 | Phloretin | 0.0000 | 1.15 | up | Flavonoids | Chalcones |
| Lmlp006175 | Isosalipurposide (Phlorizin Chalcone) | 0.0004 | -1.26 | down | Flavonoids | Chalcones |
| mws0064 | Eriodictyol (5,7,3',4'-Tetrahydroxyflavanone) | 0.0007 | 1.28 | up | Flavonoids | Flavanones |

**Supplementary Table S5.** DAMs enriched in the flavonoid biosynthesis pathway in the HE-EX vs. HE-IC comparison.

| Metabolite ID | \| Compounds \| \| --- \| | Log_2_FC | *p*-value | Direction | Class I | Class II |
| --- | --- | --- | --- | --- | --- | --- | --- |
| Hmln002806 | 5-O-Caffeoylshikimic acid | 5.58 | 0.0037 | up | Phenolic acids | Phenolic acids |
| Lmyn006227 | Galangin (3,5,7-Trihydroxyflavone) | -6.76 | 0.0011 | down | Flavonoids | Flavones |
| Lmzp002365 | Hesperetin-7-O-glucoside | -2.69 | 0.0007 | down | Flavonoids | Flavanones |
| mws0032 | Myricetin | 16.84 | 0.0001 | up | Flavonoids | Flavonols |
| mws0040 | Chrysin | -4.80 | 0.0003 | down | Flavonoids | Flavones |
| mws0042 | Epigallocatechin | 3.42 | 0.0003 | up | Flavonoids | Flavanols |
| mws0044 | Taxifolin(Dihydroquercetin) | 5.95 | 0.0004 | up | Flavonoids | Flavanonols |
| mws0046 | Naringenin-7-O-Neohesperidoside(Naringin)* | -3.15 | 0.0024 | down | Flavonoids | Flavanones |
| mws0048 | Apigenin-8-C-Glucoside (Vitexin) | -3.04 | 0.0005 | down | Flavonoids | Flavones |
| mws0049 | Gallocatechin | 2.66 | 0.0003 | up | Flavonoids | Flavanols |
| mws0789 | Pinocembrin (Dihydrochrysin) | 2.31 | 0.0009 | up | Flavonoids | Flavanones |
| mws0914 | Pinobanksin (3,5,7-Trihydroxyflavanone) | -1.19 | 0.0007 | down | Flavonoids | Flavanonols |
| mws0920 | Tricetin (5,7,3',4',5'-Pentahydroxyflavone) | -1.91 | 0.0011 | down | Flavonoids | Flavones |
| mws1068 | Kaempferol (3,5,7,4'-Tetrahydroxyflavone) | -4.38 | 0.0008 | down | Flavonoids | Flavonols |
| mws1094 | Aromadendrin (Dihydrokaempferol) | 1.79 | 0.0007 | up | Flavonoids | Flavanonols |
| mws1179 | Naringenin-7-O-glucoside (Prunin)* | -2.91 | 0.0042 | down | Flavonoids | Flavanones |
| mws2118 | Phloretin-2'-O-glucoside (Phlorizin) | 1.25 | 0.0048 | up | Flavonoids | Chalcones |
| MWSHY0048 | Dihydromyricetin (Ampelopsin) | 7.87 | 0.0017 | up | Flavonoids | Flavanonols |
| MWSHY0137 | Naringenin (5,7,4'-Trihydroxyflavanone)* | -4.04 | 0.0007 | down | Flavonoids | Flavanones |
| pmb0751 | Trans-5-O-(p-Coumaroyl)shikimate | 3.92 | 0.0612 | up | Phenolic acids | Phenolic acids |
| pmb3074 | 5-O-p-Coumaroylquinic acid* | 2.94 | 0.0020 | up | Phenolic acids | Phenolic acids |
| pme0088 | Luteolin (5,7,3',4'-Tetrahydroxyflavone) | -7.21 | 0.0064 | down | Flavonoids | Flavones |
| pme2960 | Naringenin chalcone; 2',4,4',6'-Tetrahydroxychalcone | -4.08 | 0.0006 | down | Flavonoids | Chalcones |
| pme3475 | Butin; 7,3',4'-Trihydroxyflavanone* | -4.08 | 0.0006 | down | Flavonoids | Flavanones |
| pmp000571 | Apigenin | -6.67 | 0.0001 | down | Flavonoids | Flavones |

**Supplementary Table S6.** DEGs enriched in the flavonoid biosynthesis pathway in the LE-IC vs. LE-C comparison.

| Gene ID | NR annotation | log_2_FC | *p*-value | Padj | Direction |
| --- | --- | --- | --- | --- | --- |
| LOC110631846 | flavonoid 3'-monooxygenase-like [Hevea brasiliensis] | 1.06 | 1.44672E-21 | 9.78245E-21 | up |
| LOC110632801 | omega-hydroxypalmitate O-feruloyl transferase-like [Hevea brasiliensis] | 1.40 | 3.64782E-17 | 2.01794E-16 | up |
| LOC110633409 | protein ECERIFERUM 26-like [Hevea brasiliensis] | 1.14 | 0.000393056 | 0.000780879 | up |
| LOC110633683 | probable caffeoyl-CoA O-methyltransferase At4g26220 [Hevea brasiliensis] | 1.85 | 6.43939E-44 | 8.95905E-43 | up |
| LOC110637122 | protein ECERIFERUM 2-like [Hevea brasiliensis] | -4.77 | 2.26517E-14 | 1.07683E-13 | down |
| LOC110638591 | uncharacterized protein At2g34460, chloroplastic [Hevea brasiliensis] | -1.38 | 4.43193E-06 | 1.10924E-05 | down |
| LOC110640000 | leucoanthocyanidin dioxygenase-like [Hevea brasiliensis] | -2.47 | 5.20645E-09 | 1.72084E-08 | down |
| LOC110640364 | chalcone synthase 2-like [Hevea brasiliensis] | 1.70 | 2.2507E-109 | 1.1176E-107 | up |
| LOC110641698 | caffeoyl-CoA O-methyltransferase-like [Hevea brasiliensis] | 1.22 | 3.06059E-37 | 3.54636E-36 | up |
| LOC110642702 | fatty alcohol:caffeoyl-CoA acyltransferase-like [Hevea brasiliensis] | -7.90 | 1.36522E-10 | 5.09853E-10 | down |
| LOC110643466 | anthocyanidin 3-O-glucosyltransferase 2-like [Hevea brasiliensis] | 1.77 | 1.46689E-63 | 3.18714E-62 | up |
| LOC110647282 | caffeoyl-CoA O-methyltransferase 1-like [Hevea brasiliensis] | 1.15 | 7.40495E-50 | 1.20883E-48 | up |
| LOC110647691 | vestitone reductase-like isoform X1 [Hevea brasiliensis] | 1.36 | 1.70682E-55 | 3.15606E-54 | up |
| LOC110648411 | vinorine synthase-like [Hevea brasiliensis] | 2.72 | 8.29259E-12 | 3.36622E-11 | up |
| LOC110648892 | chalcone synthase 2 [Hevea brasiliensis] | 1.08 | 2.17822E-56 | 4.09706E-55 | up |
| LOC110649288 | leucoanthocyanidin reductase-like [Hevea brasiliensis] | 1.54 | 2.66812E-85 | 8.74779E-84 | up |
| LOC110649415 | UDP-glycosyltransferase 71K1-like isoform X2 [Hevea brasiliensis] | 1.25 | 5.32188E-09 | 1.75644E-08 | up |
| LOC110649753 | anthocyanidin 3-O-glucosyltransferase 2-like [Hevea brasiliensis] | 1.18 | 3.43763E-29 | 3.07921E-28 | up |
| LOC110650078 | shikimate O-hydroxycinnamoyltransferase-like [Hevea brasiliensis] | 2.72 | 6.53604E-51 | 1.09312E-49 | up |
| LOC110651006 | protein SRG1-like [Manihot esculenta] | 3.29 | 7.60457E-55 | 1.39201E-53 | up |
| LOC110653348 | flavonoid 3',5'-hydroxylase 2-like [Hevea brasiliensis] | 1.02 | 3.53825E-45 | 5.11828E-44 | up |
| LOC110655234 | dihydroflavonol 4-reductase-like [Hevea brasiliensis] | 1.23 | 2.96183E-59 | 5.8958E-58 | up |
| LOC110658083 | anthocyanidin reductase ((2S)-flavan-3-ol-forming) [Hevea brasiliensis] | 1.25 | 3.97861E-70 | 9.86919E-69 | up |
| LOC110658204 | UDP-glycosyltransferase 88A1-like isoform X1 [Hevea brasiliensis] | 1.27 | 4.08754E-23 | 2.94473E-22 | up |
| LOC110658208 | UDP-glycosyltransferase 88A1-like [Hevea brasiliensis] | 1.22 | 0.000163862 | 0.000341985 | up |
| LOC110658723 | anthocyanidin 3-O-glucosyltransferase 1-like [Hevea brasiliensis] | -1.29 | 1.17072E-05 | 2.80076E-05 | down |
| LOC110658726 | anthocyanidin 3-O-glucosyltransferase 1-like [Hevea brasiliensis] | -1.36 | 2.24986E-10 | 8.26996E-10 | down |
| LOC110658731 | anthocyanidin 3-O-glucosyltransferase 2-like isoform X1 [Hevea brasiliensis] | 2.00 | 1.2312E-141 | 9.4986E-140 | up |
| LOC110658732 | anthocyanidin 3-O-glucosyltransferase 2-like [Hevea brasiliensis] | 1.41 | 3.62949E-75 | 9.91249E-74 | up |
| LOC110660132 | vinorine synthase-like [Hevea brasiliensis] | 2.95 | 1.00532E-42 | 1.35453E-41 | up |
| LOC110660233 | omega-hydroxypalmitate O-feruloyl transferase-like [Hevea brasiliensis] | 2.48 | 1.90302E-81 | 5.71764E-80 | up |
| LOC110660590 | flavonoid 3',5'-hydroxylase 1-like [Hevea brasiliensis] | 1.03 | 8.68394E-32 | 8.4854E-31 | up |
| LOC110662985 | protein BRI1-5 ENHANCED 1-like [Hevea brasiliensis] | 2.21 | 7.21482E-30 | 6.5747E-29 | up |
| LOC110664234 | protein SRG1-like isoform X2 [Hevea brasiliensis] | 1.54 | 7.95153E-26 | 6.36472E-25 | up |
| LOC110665236 | phenolic glucoside malonyltransferase 1-like [Hevea brasiliensis] | 1.51 | 9.21046E-20 | 5.74833E-19 | up |
| LOC110666021 | leucoanthocyanidin reductase-like [Hevea brasiliensis] | 1.99 | 2.86222E-97 | 1.14956E-95 | up |
| LOC110668325 | caffeoyl-CoA O-methyltransferase 1-like [Hevea brasiliensis] | 1.03 | 4.70788E-25 | 3.66421E-24 | up |
| LOC110668584 | protein SRG1-like [Hevea brasiliensis] | 1.42 | 9.73179E-53 | 1.70703E-51 | up |
| LOC110670394 | licodione synthase-like [Hevea brasiliensis] | -1.64 | 2.01302E-10 | 7.42337E-10 | down |
| LOC110670639 | UDP-glycosyltransferase 88A1-like [Hevea brasiliensis] | 2.13 | 9.1984E-190 | 1.3363E-187 | up |
| LOC110673296 | agmatine coumaroyltransferase-2-like [Hevea brasiliensis] | 1.62 | 7.70059E-24 | 5.69948E-23 | up |
| LOC110673929 | protein SRG1-like [Hevea brasiliensis] | 1.27 | 4.04001E-43 | 5.49486E-42 | up |
| novel.160 | cinnamoyl-CoA reductase 1-like [Hevea brasiliensis] | 3.29 | 6.77995E-15 | 3.32139E-14 | up |
| novel.191 | vestitone reductase-like isoform X1 [Hevea brasiliensis] | 1.97 | 0.000357731 | 0.000714442 | up |
| novel.2231 | protein SRG1-like [Hevea brasiliensis] | 2.34 | 5.46786E-39 | 6.63701E-38 | up |
| novel.2681 | anthocyanidin 3-O-glucosyltransferase 2-like [Hevea brasiliensis] | 1.04 | 0.001365606 | 0.002517283 | up |
| novel.2753 | UDP-glycosyltransferase 88B1 [Jatropha curcas] | -4.13 | 1.74504E-06 | 4.55107E-06 | down |
| novel.3247 | anthocyanidin 3-O-glucosyltransferase 2-like [Hevea brasiliensis] | 1.00 | 2.24286E-40 | 2.8228E-39 | up |
| novel.3845 | hypothetical protein MANES_07G031500 [Manihot esculenta] | 1.50 | 1.99099E-11 | 7.87488E-11 | up |

**Supplementary Table S7.** DEGs enriched in the flavonoid biosynthesis pathway in the HE-IC vs. HE-EC comparison.

| Gene ID | NR annotation | log_2_FC | *p-*value | Padj | Direction |
| --- | --- | --- | --- | --- | --- |
| LOC110632801 | omega-hydroxypalmitate O-feruloyl transferase-like [Hevea brasiliensis] | -1.36 | 3.90088E-21 | 3.95067E-20 | down |
| LOC110633409 | protein ECERIFERUM 26-like [Hevea brasiliensis] | 1.98 | 3.45091E-10 | 1.77573E-09 | up |
| LOC110633683 | probable caffeoyl-CoA O-methyltransferase At4g26220 [Hevea brasiliensis] | 1.24 | 0.00207335 | 0.004602443 | up |
| LOC110636257 | flavonoid 3'-monooxygenase [Hevea brasiliensis] | -1.17 | 3.84331E-43 | 8.47892E-42 | down |
| LOC110640000 | leucoanthocyanidin dioxygenase-like [Hevea brasiliensis] | -4.13 | 6.27191E-27 | 8.03911E-26 | down |
| LOC110640364 | chalcone synthase 2-like [Hevea brasiliensis] | -1.40 | 2.6848E-106 | 2.2303E-104 | down |
| LOC110640499 | BAHD acyltransferase At5g47980-like [Hevea brasiliensis] | 4.52 | 6.79276E-11 | 3.72543E-10 | up |
| LOC110641151 | naringenin,2-oxoglutarate 3-dioxygenase-like [Hevea brasiliensis] | -1.22 | 7.61418E-61 | 2.67601E-59 | down |
| LOC110641350 | chalcone synthase 1 [Hevea brasiliensis] | -1.08 | 2.70183E-80 | 1.41731E-78 | down |
| LOC110642702 | fatty alcohol:caffeoyl-CoA acyltransferase-like [Hevea brasiliensis] | -2.21 | 4.89106E-06 | 1.6214E-05 | down |
| LOC110642838 | naringenin,2-oxoglutarate 3-dioxygenase [Hevea brasiliensis] | -1.88 | 3.2404E-244 | 1.5826E-241 | down |
| LOC110643466 | anthocyanidin 3-O-glucosyltransferase 2-like [Hevea brasiliensis] | -1.63 | 2.50907E-13 | 1.63314E-12 | down |
| LOC110645776 | putative anthocyanidin reductase [Hevea brasiliensis] | -1.94 | 5.66336E-16 | 4.38682E-15 | down |
| LOC110646442 | probable 2-oxoglutarate-dependent dioxygenase At3g111800 [Hevea brasiliensis] | -1.31 | 2.09725E-09 | 9.98637E-09 | down |
| LOC110648892 | chalcone synthase 2 [Hevea brasiliensis] | -1.20 | 8.30784E-83 | 4.52569E-81 | down |
| LOC110649753 | anthocyanidin 3-O-glucosyltransferase 2-like [Hevea brasiliensis] | -2.71 | 1.84224E-42 | 4.00807E-41 | down |
| LOC110649802 | S-norcoclaurine synthase 1-like [Hevea brasiliensis] | -2.54 | 1.62397E-07 | 6.38477E-07 | down |
| LOC110650078 | shikimate O-hydroxycinnamoyltransferase-like [Hevea brasiliensis] | 1.51 | 6.99172E-22 | 7.34351E-21 | up |
| LOC110651006 | protein SRG1-like [Manihot esculenta] | 2.25 | 2.08108E-07 | 8.09207E-07 | up |
| LOC110652792 | leucoanthocyanidin dioxygenase-like [Hevea brasiliensis] | -3.07 | 4.3738E-169 | 8.6039E-167 | down |
| LOC110653493 | cytochrome P450 CYP73A100-like [Hevea brasiliensis] | -1.67 | 1.85176E-100 | 1.39137E-98 | down |
| LOC110658208 | UDP-glycosyltransferase 88A1-like [Hevea brasiliensis] | -1.38 | 5.33237E-05 | 0.000152406 | down |
| LOC110658672 | protein SRG1-like [Hevea brasiliensis] | 1.15 | 1.56831E-05 | 4.84096E-05 | up |
| LOC110658723 | anthocyanidin 3-O-glucosyltransferase 1-like [Hevea brasiliensis] | -3.05 | 1.79463E-34 | 3.00808E-33 | down |
| LOC110658726 | anthocyanidin 3-O-glucosyltransferase 1-like [Hevea brasiliensis] | -2.22 | 3.22995E-76 | 1.55868E-74 | down |
| LOC110658732 | anthocyanidin 3-O-glucosyltransferase 2-like [Hevea brasiliensis] | -1.30 | 4.73422E-29 | 6.6258E-28 | down |
| LOC110660132 | vinorine synthase-like [Hevea brasiliensis] | 4.45 | 4.86057E-70 | 2.07045E-68 | up |
| LOC110660233 | omega-hydroxypalmitate O-feruloyl transferase-like [Hevea brasiliensis] | 3.28 | 2.27872E-99 | 1.67226E-97 | up |
| LOC110667252 | vinorine synthase-like [Hevea brasiliensis] | 1.28 | 9.32937E-17 | 7.527E-16 | up |
| LOC110667357 | caffeoyl-CoA O-methyltransferase [Hevea brasiliensis] | 1.38 | 3.78508E-38 | 7.15277E-37 | up |
| LOC110668057 | probable chalcone--flavonone isomerase 3 [Hevea brasiliensis] | -1.69 | 2.4715E-124 | 2.7348E-122 | down |
| LOC110668584 | protein SRG1-like [Hevea brasiliensis] | -1.13 | 1.96855E-11 | 1.12721E-10 | down |
| LOC110670394 | licodione synthase-like [Hevea brasiliensis] | 1.02 | 3.93957E-10 | 2.01875E-09 | up |
| LOC110670575 | UDP-glycosyltransferase 88A1-like [Hevea brasiliensis] | 3.17 | 6.88189E-11 | 3.77066E-10 | up |
| LOC110673296 | agmatine coumaroyltransferase-2-like [Hevea brasiliensis] | 1.92 | 1.75194E-40 | 3.5806E-39 | up |
| novel.2231 | protein SRG1-like [Hevea brasiliensis] | 1.21 | 1.49645E-14 | 1.06107E-13 | up |
| novel.2681 | anthocyanidin 3-O-glucosyltransferase 2-like [Hevea brasiliensis] | -2.32 | 1.20462E-10 | 6.45055E-10 | down |
| novel.2753 | UDP-glycosyltransferase 88B1 [Jatropha curcas] | -4.21 | 5.87506E-26 | 7.25787E-25 | down |
| novel.3247 | anthocyanidin 3-O-glucosyltransferase 2-like [Hevea brasiliensis] | -2.38 | 2.5584E-125 | 2.8873E-123 | down |

**Supplementary Table S8.** DAMs enriched in the flavonoid biosynthesis pathway in the LE-IC vs. LE-C comparison.

| Metabolite ID | Compounds | Log_2_FC | *p*-value | Direction | Class I | Class II |
| --- | --- | --- | --- | --- | --- | --- |
| Hmln002806 | 5-O-Caffeoylshikimic acid | 2.53 | 2.64618E-05 | up | Phenolic acids | Phenolic acids |
| Lmyn006227 | Galangin (3,5,7-Trihydroxyflavone) | -3.13 | 6.06308E-07 | down | Flavonoids | Flavones |
| mws0046 | Naringenin-7-O-Neohesperidoside(Naringin)* | 1.31 | 0.006445941 | up | Flavonoids | Flavanones |
| mws0048 | Apigenin-8-C-Glucoside (Vitexin) | -3.73 | 0.006867526 | down | Flavonoids | Flavones |
| mws0064 | Eriodictyol (5,7,3',4'-Tetrahydroxyflavanone) | 1.48 | 0.000259639 | up | Flavonoids | Flavanones |
| mws0789 | Pinocembrin (Dihydrochrysin) | 1.03 | 3.94616E-05 | up | Flavonoids | Flavanones |
| MWSHY0048 | Dihydromyricetin (Ampelopsin) | 2.77 | 0.001043615 | up | Flavonoids | Flavanonols |

| Metabolite ID | Compounds | Log_2_FC | *p*-value | Direction | Class I | Class II |
| --- | --- | --- | --- | --- | --- | --- |
| mws0044 | Taxifolin(Dihydroquercetin) | -1.15 | 6.61531E-05 | down | Flavonoids | Flavanonols |
| mws0049 | Gallocatechin | 1.10 | 3.22303E-05 | up | Flavonoids | Flavanols |
| MWSHY0137 | Naringenin (5,7,4'-Trihydroxyflavanone)* | -1.69 | 1.97284E-05 | down | Flavonoids | Flavanones |
| pmb0751 | Trans-5-O-(p-Coumaroyl)shikimate | 2.30 | 0.002220656 | up | Phenolic acids | Phenolic acids |
| pmb3074 | 5-O-p-Coumaroylquinic acid* | 1.24 | 0.009577303 | up | Phenolic acids | Phenolic acids |
| pme2960 | Naringenin chalcone; 2',4,4',6'-Tetrahydroxychalcone | -1.70 | 1.38508E-05 | down | Flavonoids | Chalcones |
| pme3475 | Butin; 7,3',4'-Trihydroxyflavanone* | -1.66 | 2.9254E-05 | down | Flavonoids | Flavanones |

**Supplementary Table S9.** DAMs enriched in the flavonoid biosynthesis pathway in the HE-IC vs. HE-EC comparison

**
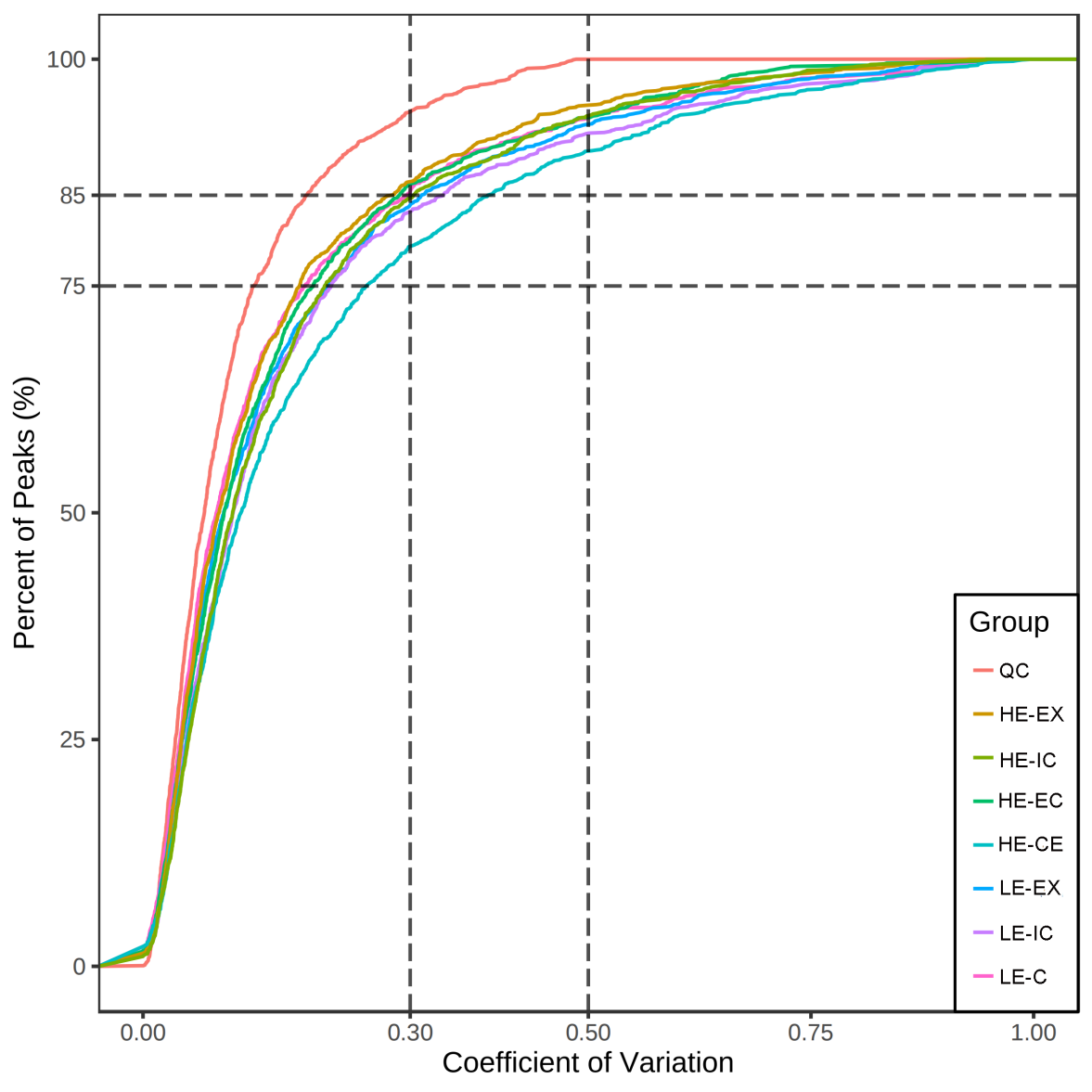
**

**Supplementary Figure S1.** Distribution of coefficient of variation (CV) values across all samples. This figure shows the empirical cumulative distribution of CV values for all detected metabolites across sample groups. The x-axis represents the CV value, and the y-axis shows the proportion of metabolites with CV values less than or equal to the corresponding value. Different colors denote different sample groups, with QC representing quality control samples. Vertical reference lines mark CV values of 0.3 and 0.5, and horizontal reference lines indicate proportions of 75% and 85% of the total metabolites.


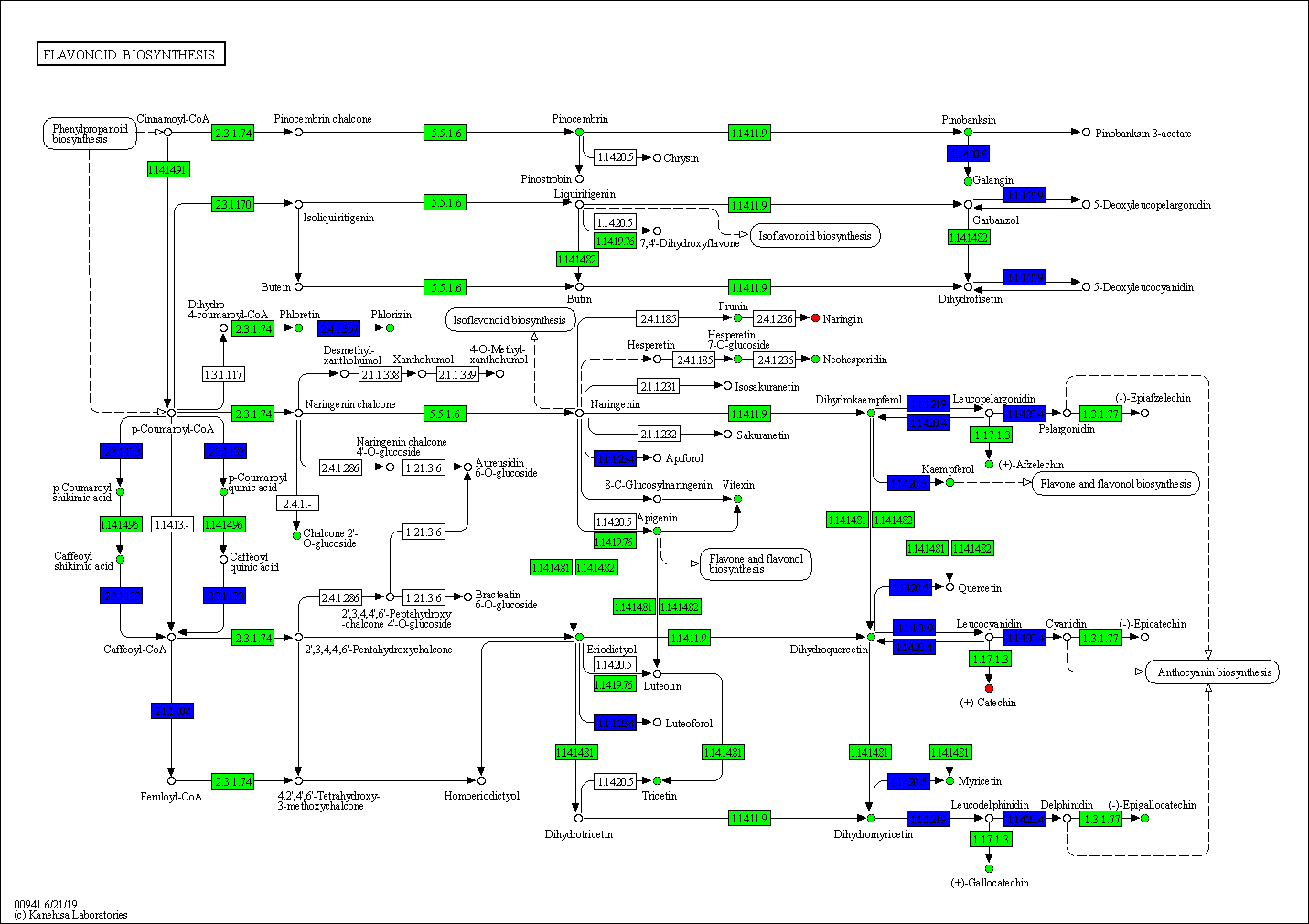


**Supplementary Figure S2.** Integrated KEGG pathway visualization of flavonoid biosynthesis. This figure shows DEGs and DAMs enriched in the flavonoid biosynthesis pathway in the HE-EC vs. HE-CE comparison. Circles represent metabolites, and rectangles represent genes. Red and green indicate upregulated and downregulated genes/metabolites, respectively, while blue indicates pathways containing both.


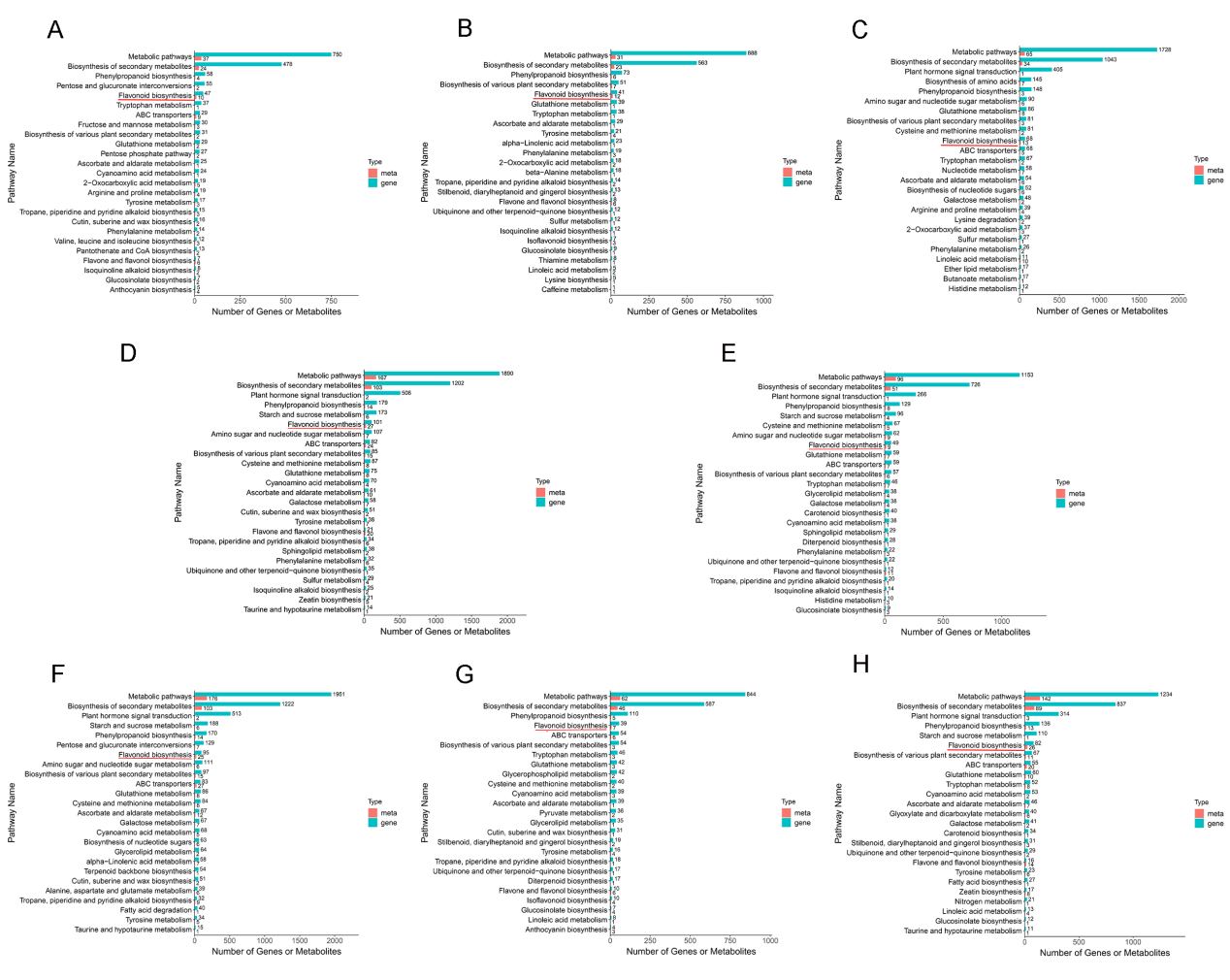


**Supplementary Figure S3.** KEGG enrichment analysis of DEGs and DAMs. This figure presents KEGG pathway enrichment jointly identified in transcriptomic and metabolomic datasets. Only pathways enriched in both omics layers were included; when more than 25 pathways were shared, the top 25 based on transcriptome-derived P-values were shown. The x-axis indicates the number of DEGs and DAMs in each pathway, and the y-axis lists KEGG pathway names. Red and green bars represent metabolomic and transcriptomic enrichment, respectively. Comparisons are shown in panels **(A)** LE-EX vs. HE-EX, **(B)** LE-IC vs. HE-IC, **(C)** LE-C vs. HE-EC, **(D)** LE-EX vs. LE-IC, **(E)** LE-IC vs. LE-C, **(F)** HE-EX vs. HE-IC, **(G)** HE-IC vs. HE-EC, and **(H)** HE-EC vs. HE-CE.


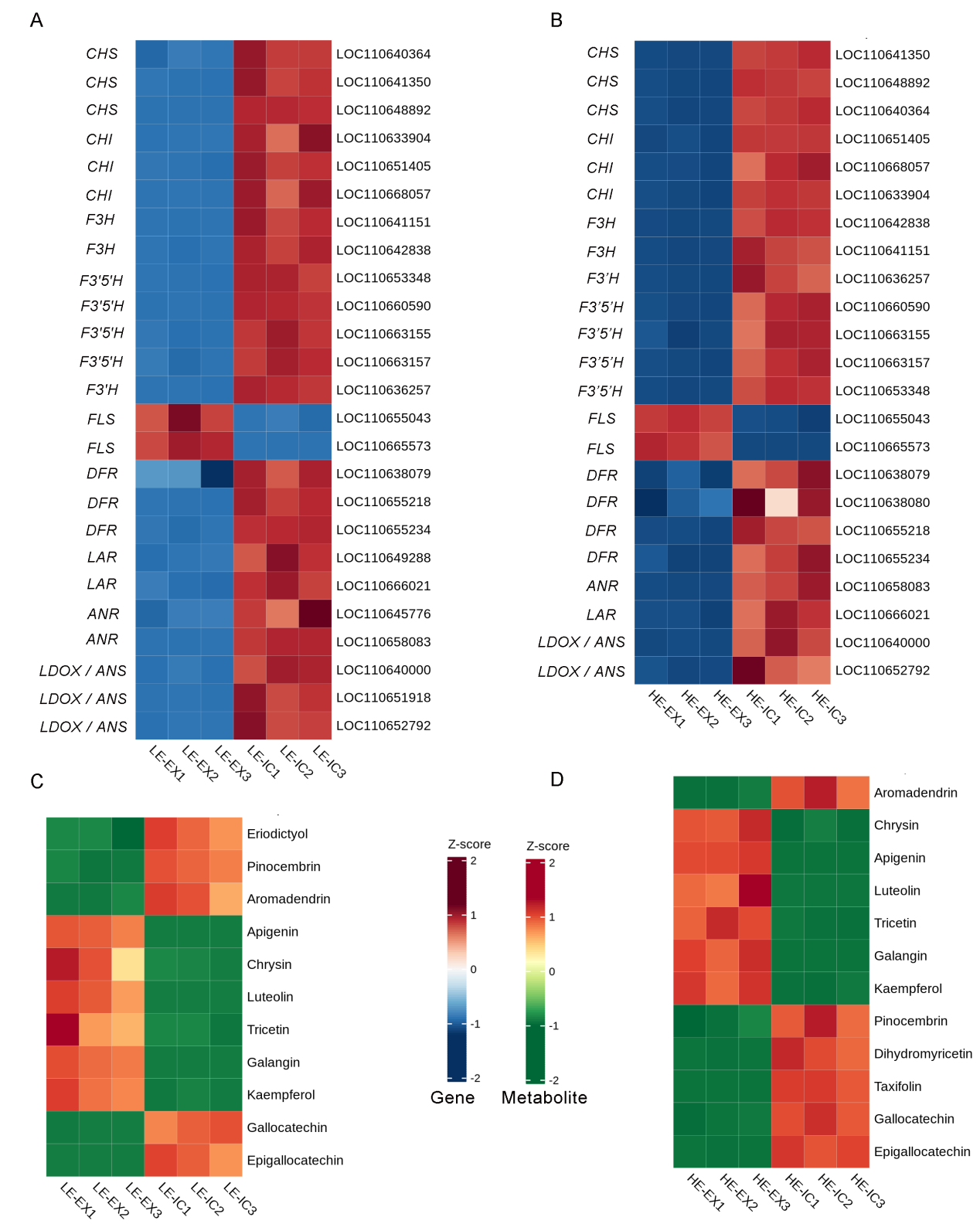


**Supplementary Figure S4.** Replicate-level heatmaps supporting the flavonoid pathway analysis in Figure 6. Detailed heatmaps showing individual replicate data for differentially expressed genes and metabolites in the flavonoid biosynthesis pathway during callus induction. **(A)** Gene expression heatmap for LE-EX vs. LE-IC comparison (n=3 biological replicates per stage). **(B)** Gene expression heatmap for HE-EX vs. HE-IC comparison (n=3 biological replicates per stage). **(C)** Metabolite abundance heatmap for LE-EX vs. LE-IC comparison. **(D)** Metabolite abundance heatmap for HE-EX vs. HE-IC comparison. Values are Z-score normalized. Gene expression: blue (low) to red (high). Metabolite abundance: green (low) to red (high). This supplementary figure provides the replicate-level quantitative data underlying the integrated pathway visualization presented in Figure 6.


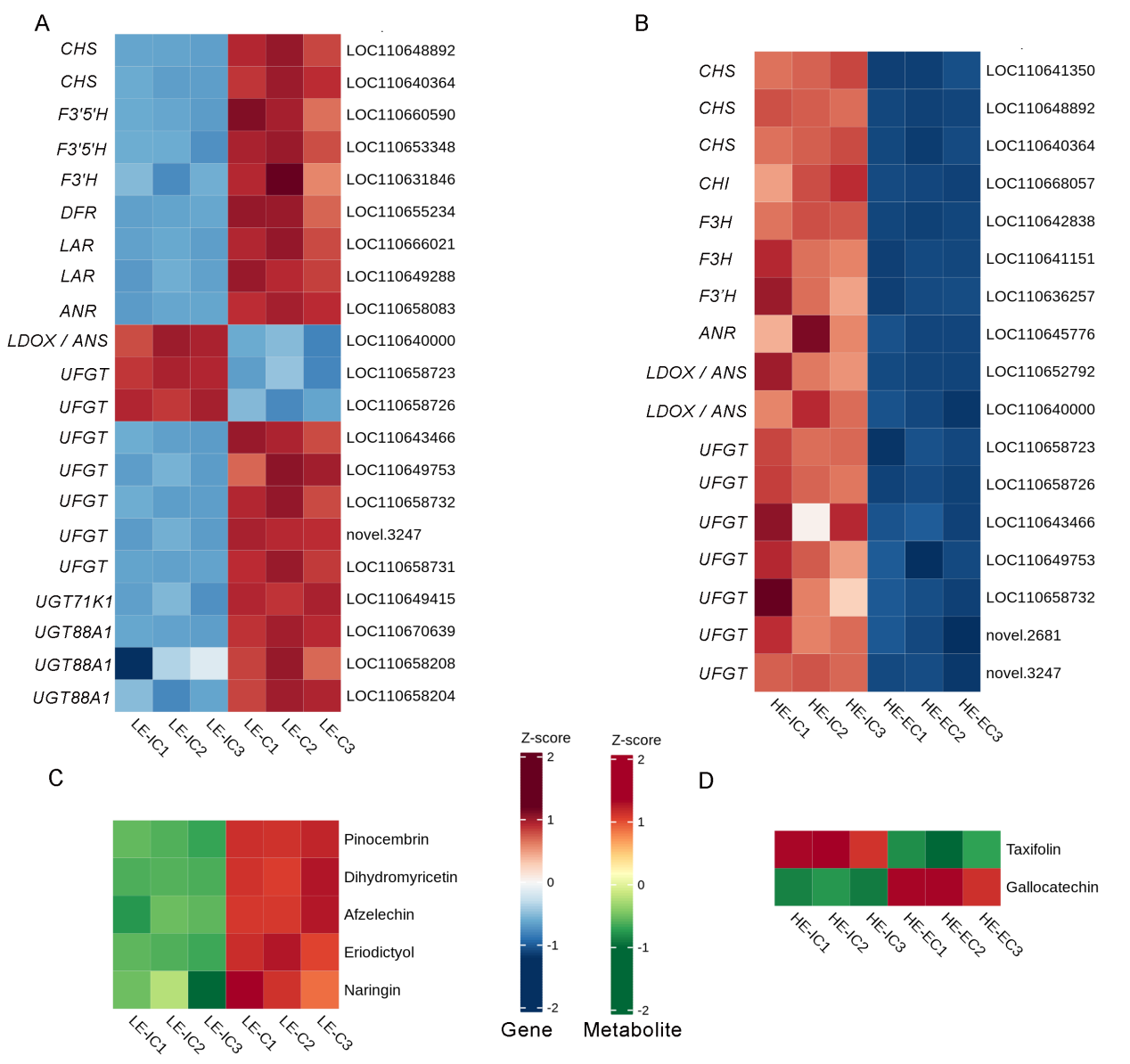


**Supplementary Figure S5.** Replicate-level heatmaps supporting the flavonoid pathway analysis during differentiation in Figure 8. Detailed heatmaps showing individual replicate data for differentially expressed genes and metabolites in the flavonoid biosynthesis pathway during the callus-to-differentiation transition. **(A)** Gene expression heatmap for LE-IC vs. LE-C comparison (n=3 biological replicates per stage). **(B)** Gene expression heatmap for HE-IC vs. HE-EC comparison (n=3 biological replicates per stage). **(C)** Metabolite abundance heatmap for LE-IC vs. LE-C comparison. **(D)** Metabolite abundance heatmap for HE-IC vs. HE-EC comparison. Values are Z-score normalized. Gene expression: blue (low) to red (high). Metabolite abundance: green (low) to red (high). This supplementary figure provides the replicate-level quantitative data underlying the integrated pathway visualization presented in Figure 8, which demonstrates divergent metabolic regulation strategies (maintenance-type in LE vs. conversion-type in HE) during somatic embryo differentiation.
